# Evaluation of the lasso and the elastic net in genome-wide association studies

**DOI:** 10.3389/fgene.2013.00270

**Published:** 2013-12-04

**Authors:** Patrik Waldmann, Gábor Mészáros, Birgit Gredler, Christian Fuerst, Johann Sölkner

**Affiliations:** ^1^Division of Livestock Sciences, Department of Sustainable Agricultural Systems, University of Natural Resources and Life SciencesVienna, Austria; ^2^Division of Statistics, Department of Computer and Information Science, Linköping UniversityLinköping, Sweden; ^3^Qualitas AGZug, Switzerland; ^4^ZuchtData EDV-Dienstleistungen GmbHVienna, Austria

**Keywords:** lasso, elastic net, simulation, GWAS, population structure, cattle

## Abstract

The number of publications performing genome-wide association studies (GWAS) has increased dramatically. Penalized regression approaches have been developed to overcome the challenges caused by the high dimensional data, but these methods are relatively new in the GWAS field. In this study we have compared the statistical performance of two methods (the least absolute shrinkage and selection operator—lasso and the elastic net) on two simulated data sets and one real data set from a 50 K genome-wide single nucleotide polymorphism (SNP) panel of 5570 Fleckvieh bulls. The first simulated data set displays moderate to high linkage disequilibrium between SNPs, whereas the second simulated data set from the QTLMAS 2010 workshop is biologically more complex. We used cross-validation to find the optimal value of regularization parameter λ with both minimum MSE and minimum MSE + 1SE of minimum MSE. The optimal λ values were used for variable selection. Based on the first simulated data, we found that the minMSE in general picked up too many SNPs. At minMSE + 1SE, the lasso didn't acquire any false positives, but selected too few correct SNPs. The elastic net provided the best compromise between few false positives and many correct selections when the penalty weight α was around 0.1. However, in our simulation setting, this α value didn't result in the lowest minMSE + 1SE. The number of selected SNPs from the QTLMAS 2010 data was after correction for population structure 82 and 161 for the lasso and the elastic net, respectively. In the Fleckvieh data set after population structure correction lasso and the elastic net identified from 1291 to 1966 important SNPs for milk fat content, with major peaks on chromosomes 5, 14, 15, and 20. Hence, we can conclude that it is important to analyze GWAS data with both the lasso and the elastic net and an alternative tuning criterion to minimum MSE is needed for variable selection.

## Introduction

The genome-wide association study (GWAS) is a well-established technique for identifying genetic variants of interest, not only for many common complex human diseases but also for traits of interest in animal and plant genetics (McCarthy et al., 2008; Goddard and Hayes, 2009; Heffner et al., 2009). While the GWAS approach is extensively used and generally celebrated, there are still several unresolved statistical challenges in studying the joint effects of that huge number of genetic and environmental variables (Cantor et al., 2010; Moore et al., 2010). First, in a typical GWAS, genotypes from some thousands to several millions single nucleotide polymorphism (SNP) markers are determined in subjects in the order of a few thousands, leading to the small *n*, large *p* problem (i.e., many more predictor variables than response variables). It is common in GWAS to perform single SNP regression which leads to very high rates of Type I error (false positives). The standard procedure is then to adjust the significance threshold with some kind of multiple comparison criteria, for example Bonferroni or False Discovery Rate (FDR) corrections. Unfortunately, these corrections will unavoidably introduce Type II errors (false negatives) and true SNP-associations of moderate and small effects will be erroneously discarded. Therefore, one of the most important issues in contemporary statistical genetics is to find methods that provide a satisfactory balance between false positives and false negatives in large-scale GWAS (Rice et al., 2008). Secondly, when a large number of SNPs are genotyped on a genome-wide scale, correlated variables (linkage disequilibrium between SNPs) occur both because of biological factors (e.g., population history) and because of high dimensionality (Fan and Lv, 2010).

There are several applications of regularized regression methods to GWAS (reviewed in Szymczak et al., 2009; Dasgupta et al., 2011) and genomic selection, which is closely related to GWAS but concerned with prediction of genomic breeding values (reviewed in de Los Campos et al., 2013). While in genomic selection the focus is on predicting future performance, the association studies are designed to find genetic makers connected to the trait of interest. Penalized regression methods have been previously used for variable selection in high dimensional studies focused on human genetic data (e.g., Sung et al., 2009; Wu et al., 2009; Cho et al., 2010). Kooperberg et al. (2010) compared the performance of elastic net and lasso using uncorrelated predictor variables. Ayers and Cordell (2010) examined the influence of penalties in several penalized regression models.

To overcome the challenges mentioned above, penalized regression approaches, also called shrinkage or regularization methods, have been developed. Although shrinking some of the regression coefficients toward zero may result in biased estimates, these regression coefficient estimates will have smaller variance. This can result in enhanced prediction accuracy because of a smaller mean squared error (Hastie et al., 2009). Regression coefficients are shrunk by imposing a penalty on their size, which is done by adding a penalty function to the least-squares model. Moreover, some of these procedures (e.g., the lasso) enable variable selection such that only the important predictor variables stay in the model (Szymczak et al., 2009).

Tibshirani (1996) proposed the lasso estimator which estimates the regression coefficients through an ℓ_1_-norm penalized least-squares criterion. This is equivalent to minimizing the sums of squares of residuals plus an ℓ_1_ penalty on the regression coefficients. While demonstrating promising performance for many problems, the lasso estimator does have some shortcomings (Zou and Hastie, 2005). Firstly, the lasso tends to have problems when predictor variables are highly correlated. In the extreme case of *k* identical predictor variables, the lasso breaks down. Secondly, when there is some group or cluster structure among the predictor variables, the lasso estimator usually selects only one predictor from a group while ignoring others. Thirdly, the lasso method cannot select more predictor variables than the sample size. This could potentially be a problem in various genomic studies that involve many more, often highly correlated, predictor variables than response variables. From a Bayesian point of view, the lasso penalty corresponds to a Laplace (double exponential) prior over the regression coefficients, which expects many coefficients to be close to zero, and a small subset to be larger and non-zero (Kyung et al., 2010). However, the Bayesian lasso doesn't set any variables to exactly zero and therefore needs to be combined with some other form of variable selection (Hans, 2010).

On the other hand, ridge regression (Hoerl and Kennard, 1970) estimates the regression coefficients through an ℓ_2_-norm penalized least-squares criterion. It is well-known that ridge regression shrinks the coefficients of correlated predictor variables toward each other, allowing them to borrow strength from each other (Friedman et al., 2010). However, this behavior is not without its problems. For example, in the case of the *k* identical predictor variables mentioned above, they each get identical coefficients with 1/*k* the size that any single one would get if fit alone. The ridge penalty is ideal if there are many predictor variables, and all have non-zero coefficients (from a Bayesian perspective these are drawn from a Gaussian prior distribution). One important difference between the lasso and ridge regression occurs for the predictor variables with the highest regression coefficients. Whereas the ℓ_2_ penalty pushes the regression coefficients toward zero with a force proportional to the value of the coefficient, the ℓ_1_ penalty exerts the same force on all non-zero coefficients. Hence for the variables that are most valuable (i.e., that clearly should be in the model and where shrinkage toward zero is less desirable) an ℓ_1_ penalty shrinks less (Hesterberg et al., 2008). The extent of shrinkage in ridge regression is also dependent on allele frequency and sample size (Gianola, 2013).

Due to the drawbacks of using the lasso and ridge regression on their own, Zou and Hastie (2005) proposed the elastic net penalty which is based on a combined penalty of lasso and ridge regression penalties. The penalty parameter α determines how much weight should be given to either the lasso or ridge regression. The elastic net with α set to 0 is equivalent to ridge regression. The elastic net with α close to 1 performs much like the lasso, but removes any degeneracies and odd behavior caused by high correlations. Studies have shown that analysis with the elastic net can result in lower mean squared errors than the lasso and ridge regression when predictor variables are correlated (Bühlmann and van de Geer, 2011). Moreover, the elastic net produces a higher number of correctly identified influential variables than the lasso, and has much lower false positive rate than ridge regression (Tutz and Ulbricht, 2009).

The purpose of this study is to compare the statistical properties of the lasso and the elastic net in a typical GWA framework. Ridge regression was not tested because it selects all variables. The analyses are performed with the R package glmnet which uses the fast cyclical coordinate descent (CCD) algorithm (Friedman et al., 2010). Tests are performed on simulated SNP data that exhibit LD, on simulated pedigree data from the QTLMAS 2010 workshop (Szydłowski and Paczyñska, 2011) as well as on real data from a 50 K genome-wide SNP panel of 5570 individuals in a dairy cattle study on fat content in milk.

## Materials and methods

### Methods

Consider a standard multiple linear regression model

(1) $$y=1\beta_{0}+\text{X}\beta +e$$

where *y* is a vector of length *n* including the response variable, X = (*x*_*i*1_,…,*x*_*ip*_) is a *n* × *p* matrix holding the predictor variables, β_0_ is the intercept, β = (β_1_, …,β_*p*_) is a column vector that contains the regression coefficients and *e* is a vector of error terms assuming normal distribution *e* ~ *N* (0, σ^2^_*e*_). For models where *n* > *p*, the values of the unknown parameters β_0_ and β can be uniquely estimated by minimizing the residual sum of squares

(2) $${\hat{\beta }}_{0},\hat{\beta }=\underset{\beta_{0},\;\beta }{\text{argmin}}\sum_{i=1}^{n}{\left(y_{i}-\beta_{0}-\sum_{j=1}^{p}\beta_{j}X_{ij}\right)}^{2}.$$

The number of SNPs (coded either as 0, 1, 2 or −1, 0, 1) is generally much larger than the number of observations in a typical GWAS. A penalized regression function is formulated as

(3) $${\hat{\beta }}_{0},\hat{\beta }=\underset{\beta_{0},\;\beta }{\text{argmin}}\left[\sum_{i=1}^{n}{\left(y_{i}-\beta_{0}-\sum_{j=1}^{p}\beta_{j}X_{ij}\right)}^{2}+\text{P}\left(\lambda ,\;\beta \right)\right]$$

where *P* (λ, β) a general penalty function with regularization parameter λ. The lasso penalty (Tibshirani, 1996) regularizes the linear regression coefficients through an ℓ_1_-norm penalized least-squares criterion [i.e., *P* (λ, β) = λ ‖β‖_ℓ_1__]

(4) $${\hat{\beta }}_{0},\hat{\beta }=\underset{\beta_{0},\;\beta }{\text{argmin}}\left[\sum_{i=1}^{n}{\left(y_{i}-\beta_{0}-\sum_{j=1}^{p}\beta_{j}X_{ij}\right)}^{2}+\lambda \sum_{j=1}^{p}\left|\beta_{j}\right|\right]$$

The resulting regression problem is non-linear in *y* and results in a convex optimization problem. The regularization parameter λ controls the amount of shrinkage and needs to be tuned or chosen based on some prior results.

Ridge regression (Hoerl and Kennard, 1970) estimates the linear regression coefficients through an ℓ_2_-norm penalized least-squares criterion [i.e., *P* (λ, β) = λ ‖β‖_ℓ_2__]

(5) $${\hat{\beta }}_{0},\hat{\beta }=\underset{\beta_{0},\;\beta }{\text{argmin}}\left[\sum_{i=1}^{n}{\left(y_{i}-\beta_{0}-\sum_{j=1}^{\text{p}}\beta_{j}{\text{X}}_{ij}\right)}^{2}+\lambda \overset{\text{p}}{\underset{j=1}{\sum }}\beta_{j}^{2}\right]$$

This minimization problem can be solved analytically. In ridge regression, the regularization parameter λ controls the amount of shrinkage, but no predictor variables are set to zero. The shrinkage makes the β estimates biased but with a smaller variance. The regularization also facilitates the conditioning of the underdetermined system of linear equations that occurs in the *p* >> *n* situation, thus enabling a numerical solution. For correlated predictor variables, ridge regression shrinks the coefficients toward each other.

The elastic net (EN) method (Zou and Hastie, 2005) is based on a compromise between the lasso and ridge regression penalties

(6) $$\begin{array}{l} {\hat{\beta }}_{0},\hat{\beta }=\underset{\beta_{0},\;\beta }{\text{argmin}}\{\sum_{i=1}^{n}{\left(y_{i}-\beta_{0}-\sum_{j=1}^{p}\beta_{j}X_{ij}\right)}^{2}\\ +\lambda \sum_{j=1}^{p}\left[\frac{1}{2}\left(1-\alpha \right)\beta_{j}^{2}+\alpha \left|\beta_{\text{j}}\right|\right]\} \end{array}$$

where 0 ≤ α ≤ 1 is a penalty weight. The EN with α = 1 is identical to the lasso, whereas it turns out to be ridge regression with α = 0 (Friedman et al., 2010). Setting α close to 1 makes the EN to behave similar to the lasso, but eliminates problematic behavior caused by high correlations. When α increases from 0 to 1, for a given λ the sparsity of the minimization (i.e., the number of coefficients equal to zero) increases monotonically from 0 to the sparsity of the lasso estimation. The elastic net can select more variables than observations.

Recently, Friedman et al. (2007, 2010) developed a computationally efficient cyclic coordinate descent (CCD) method for estimation and prediction in regression models with lasso, ridge regression and the elastic net penalties. The CCD method is similar to the forward, stepwise multiple regression approach. First, it finds the λ_max_ value along the regularization path for which the entire vector $\hat{\beta }=0$. The strategy is then to select a minimum value λ_min_ = ελ_max_, and construct a sequence of *K*-values of λ decreasing from λ_max_ to λ_min_ on the log scale. Typical values are ε = 0.001 and *K* = 100. Variables are then added iteratively according to their importance along the λ path. One iteration in the CCD algorithm is based on first computing the simple least-squares coefficient on the partial residual, then applying soft thresholding to take care of the lasso contribution to the penalty, and finally using a proportional shrinkage for the ridge penalty. We refer to Friedman et al. (2007, 2010) for details regarding the algorithm, but we note here that there are options for *naïve* updates, where a complete cycle through all *p* variables has a computational cost of *O*(*pn*) operations, as well as for *covariance* updates, with *m* non-zero terms in the model, where a complete cycle costs *O*(*pm*) operations if no new variables become non-zero and *O*(*pn*) for each new variable entered. The covariance updating algorithm is more efficient for small data sets (*p* < 500), whereas the naïve updating is more efficient when *n* ≪ *p*.

The optimal value of λ, where the predictor selection should be done, can then be found by *k*-fold cross-validation to find the minimum mean squared error (minMSE) or minMSE + 1 standard error of minMSE backwards along the λ path (minMSE + 1SE), i.e., the largest λ-value such that the error is within 1SE of the minimum. In *k*-fold cross-validation, the original sample is randomly partitioned into *k* subsamples. One subsample is then taken as the validation data for testing the model, and the remaining *k* − 1 subsamples are used as training data. The cross-validation procedure is repeated *k* times (the number of folds), with each of the *k* subsamples used only once as the validation data. The *k* results from the folds are then averaged to create a single estimation with standard errors. The methods above have been implemented in an R package called glmnet (Friedman et al., 2010). minMSE and minMSE + 1SE are automatically calculated using arguments s = “lambda.min” and s = “lambda.1se” in the cv.glmnet function.

Single SNP regression is common in GWAS, but the significance tests need to be adjusted for multiple comparison. We performed one single regression analysis for each SNP on all data sets and collected the corresponding *p*-values in vectors. We then calculated the local false discovery rate following Efron (2010)

(7) $$\text{fdr}\left(z\right)=Pr\left(“\text{null}”|z\right)=\frac{\pi_{0}f_{0}\left(z\right)}{f\left(z\right)},$$

where *f* (*z*) is a mixture density

(8) $$f\left(z\right)=\pi_{0}f_{0}\left(z\right)+\pi_{1}f_{1}\left(z\right),$$

*z*_*i*_ = Φ^−1^ (*p*_*i*_) is the inverse cumulative normal distribution function of the *p*-values, π_0_ = Pr {null} and π_1_ = Pr {non − null} (the prior probabilities of the null and non-null hypotheses), *f*_0_ (*z*) the null density and *f*_1_ (*z*) the non-null density. Estimates of fdr(*z*) can be obtained by

(9) $$\hat{fdr}\left(z\right)=\frac{{\hat{\pi }}_{0}{\hat{f}}_{0}\left(z\right)}{\hat{f}\left(z\right)}$$

where $\hat{f}\left(z\right)$ is estimated by Poisson mixture regression of the *z*-values (section 5.2 in Efron, 2010). The mean and variance of the empirical null ${\hat{f}}_{0}\left(z\right)\;\sim \;N\left(\delta_{0},\sigma_{0}^{2}\right)$ are together with ${\hat{\pi }}_{0}$ also obtained from the *z*-values, but with Maximum Likelihood Estimation according to section 6.3 in Efron (2010). We used the locfdr (Efron et al., 2011) R package for estimating fdr(*z*) and set a threshold value of 0.2 according to recommendations (Efron, 2010).

### Simulated data

The purpose of the first simulation setting was to evaluate how different levels of correlation (LD) between the predictor variables (SNP genotypes) influence the statistical properties of the lasso, ridge regression and the elastic net with varying values of α. Three correlation settings were considered using a data set of 1000 records and 25 important predictor variables (SNPs) selected out of a total of 50,000 predictor variables. These 25 predictor variables were divided into blocks of 5 and centered at position 1000, 10,000, 20,000, 30,000, and 40,000, respectively. In the first correlation setting, all predictor variables should display high correlation with each other, both within and between groups. The second setting was designed for a mixture between high and medium correlations, and the third contained only medium correlations between all predictor variables. The selected positions for important variables had no meaning in terms of chromosomal locations, any position selected on purpose or random would lead to the same result of correctly or incorrectly identified predictor variables. The R script to generate and analyze the data is provided as a supplement to this paper.

The data for the first setting was simulated as follows: First, we generated 1000 continuous values (phenotypes), where the first half was associated with high **y**_[1:500]_ ~ *N* (2, 1) and the second half with low **y**_[501:1000]_ ~ *N* (−2, 1) values without additional sorting of phenotypes. The genotypes were constructed by randomly simulating values (0 or 1) from a binomial distribution with frequency 0.5 into two matrices of size 1000 × 50,000. The numbers of these matrices were then added to obtain SNP genotypes (predictor variables) coded as 0, 1, and 2 which is common practice in GWAS. In order to get high effects and correlations of the 25 selected predictor variables, these were simply sorted, independently from each other, so that all SNP genotypes 2 and half of genotype 1 were associated with the higher sorted phenotypic values. Accordingly, the other half of genotypes 1 and all genotypes 0 were associated with low values. This procedure resulted in an average correlation coefficient, *r*, between the 25 important predictor variables of 0.97 (*r*^2^ = 0.94).

In the second correlation setting (with a mixture of high and medium correlations), we used the data from the first setting and randomly permuted 25% of the sorted values within each of the 5 predictor variables centered around positions 1000 and 40,000 (i.e., resulting in a total of 10 predictor variables with medium correlations and 15 with high correlations). The average correlation between these 25 predictor variables was 0.78 (*r*^2^ = 0.62). In the third correlation setting (with only medium correlations), we randomly permuted 25% of the sorted values within each of all the 25 important predictor variables. This resulted in a mean correlation between these 25 predictor variables of 0.55 (*r*^2^ = 0.30). A hundred replicates were generated for each of the three correlation settings. One smaller (5000 predictor variables) and one larger (100,000 predictor variables) high correlation data set was also produced to test the influence of the ratio between *n* and *p* on α. Finally, in order to evaluate the influence of number of important predictor variables on α, we generated 100 replicates of the high correlation setting with 100 important predictor variables to be selected.

### QTLMAS 2010 data

This data was initially constructed for the QTLMAS 2010 workshop (Szydłowski and Paczyñska, 2011). The number of individuals in the simulated pedigree was 3226 individuals of 5 generations deep. Of the 20 founders 5 were males and 15 were females. The pedigree structure was created assuming that each female mates once (mainly with males from their own generation) and gives birth to approximately 30 progeny. Five autosomal chromosomes were simulated, each about 100 Mbp long. The biallelic SNP data was simulated using a neutral coalescent model. The genomes for founders were compiled by drawing a pair of haplotypes from the haplotype pool. The founders' alleles were then dropped down the pedigree with a recombination rate of 1 cM/Mb and a mutation rate of 10^−8^ per base per generation. The simulation algorithm produced 10,031 markers, including 263 monomorphic and 9768 biallelic SNPs. Out of the 9345 SNPs with *MAF* >0.05, 3933 loci showed significant deviation from Hardy–Weinberg equilibrium (Pearson test under individual test error rate of 1%). Mean LD (*r*^2^ calculated from unphased genotypes) between adjacent SNPs with *MAF* >0.05 was 0.100 (*SD* = 0.152).

The continuous quantitative trait used in our study was determined by 37 QTLs, including 9 controlled genes and 28 random genes. The controlled genes were selected based on their high polymorphism and high LD with markers. The random genes were drawn from the simulated SNPs (excluding chromosome 5), whereas their additive effects were sampled from a truncated normal distribution, *N*(0, 10), and then accepted if abs(add. eff.) <2. Each simulated QTL was surrounded by 19–47 polymorphic SNPs (*MAF* >0.05) located within 1 Mb distance from the QTL. Of these, 364 SNPs were in moderate to high LD with the QTLs (*r*^2^ > 0.1). Residuals were assumed to be uncorrelated and sampled from a normal distribution with variance of 51.76. The narrow-sense heritability (*h*^2^) was 0.52 for males and 0.39 for females. For data analyses SNPs with *MAF* <0.01 were discarded, leaving a final sample of 9723 SNPs. We did not check for SNPs that deviate from HWE because we did not want to lose loci that could be under selection or in LD with selected QTLs.

### Cattle data

Deregressed breeding values (Garrick et al., 2009) for milk fat content were calculated for dual purpose Fleckvieh bulls from the joint German-Austrian genomic evaluation. Genotypes of the bulls were scored as 0, 1, and 2 based on data from the Illumina bovine 54 K SNP chip, but only unambiguously mapped SNPs based on Fadista and Bendixen (2012) were kept for further analysis. The quality of genotypic data was checked for minimum call rate >90% and *MAF* >1%. After correction for missing genotypes and phenotypes, the number of bulls was 5570. Missing alleles were replaced with the average allele frequency. SNPs significantly deviating from the Hardy–Weinberg equilibrium (*p* < 0.00001) were deleted. Hence, a total of 34,373 SNPs were used in the final analyses.

### Estimation of population structure using spectral graphs

Population structure can lead to spurious false positive associations in GWAS. There are several techniques available to adjust for population structure (Price et al., 2010; Sillanpää, 2011). We have chosen a new technique based on spectral graph theory that is related to the popular PCA method (Lee et al., 2010). The main goal of the spectral graph approach is to estimate a significant number of eigenvectors from the genomic markers (SNPs) that can be used as fixed covariates in the penalized regression analyses. The underlying idea of this technique is to represent the population as a weighted graph, where the vertex set is comprised by the individuals in the study, and the weights reflect the degree of genetic similarity between pairs of subjects. The graph is then embedded in a lower dimensional space using the top eigenvectors of a function of the weight matrix. The number of significant eigenvectors can then be estimated by a simulation approach that generates a reference population without population structure, for details see Lee et al. (2010). We used the methods implemented in the R package GemTools (Klei et al., 2011) for the QTLMAS 2010 and cattle data. The eigenvectors enter the glmnet model without any penalty, using the penalty.factor function argument.

## Results

### Simulated data

**Figure 1:**
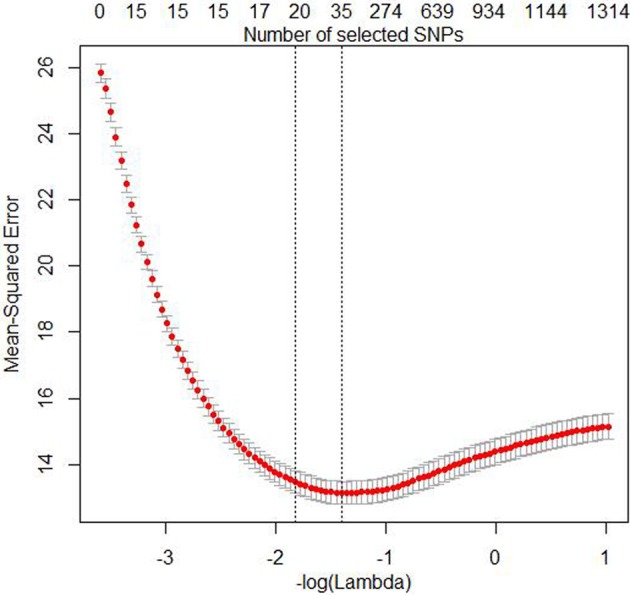
**Plot of the Mean-Squared Error (MSE) and the number of SNPs in the model as functions of -log( λ) for the 10-fold cross-validation analyses with the EN01 penalty of one the mixed LD simulated data sets**. The red dots are the mean form the cross-validation and the bars indicate mean + 1SE and mean **−** 1SE, respectively. The minMSE + 1SE of minMSE (left) and minMSE (right) and are indicated by the dashed vertical lines.

The three simulation settings were evaluated at the following penalty weights (α = 1, 0.75, 0.5, 0.3, 0.1, 0.05, 0.01), where α = 1 is equivalent to the lasso. Ridge regression is obtained with α = 0, but we did not perform any analyses with this value because ridge regression doesn't perform any variable selection. All other α are elastic net penalties with varying degree of ℓ_1_- and ℓ_2_-norm influence. The optimal value of λ (i.e., the stopping criterion) was obtained by performing 10-fold cross-validations to find the minMSE value and the minMSE + 1SE value. Friedman et al. (2010) recommend using the latter because this avoids over fitting, although we report results for both here. Figure 1 shows both the number of SNPs in the model and MSE as functions of λ. The figure also shows where the minMSE and minMSE + 1SE are located.

We note that λ at minMSE provides results with too many selected predictor variables because all evaluated models yielded relatively large levels of false positives under all three correlation settings (Table 1). Another interesting result for this stopping criterion is that the lasso and some elastic net procedures with high α values start to overfit (based on number of false positives) without finding the correct number of predictor variables. For the minMSE + 1SE stopping criterion, the lasso never picked up any false positives, but acquired too few correct predictor variables for all three simulation settings, with a median of only 3 and 2 correct predictor variables out of 25 for the high and mixed correlations, respectively (Table 1). The elastic net generally performed better, with α = 0.1 and 0.05 resulting in no missing predictor variables and no false positives for the high and low correlation setting. In the mixed correlation setting, no method yielded a perfect result, but α = 0.05 picked up 24 out of the 25 predictor variables and had no false positives. The 5000 and 100,000 predictor data sets both produced results that were very similar to the 50,000 predictor setting (results not shown). For the data with 100 important predictor variables, the lasso selected a median of 4 correct predictor variables and 0 false positives. The elastic net with α = 0.01 selected a median of 100 correct and 0 false positives, whereas α = 0.05 resulted in a median of 97 correct and 0 false positives. This is a similar finding as for the 25 predictor variables in the previous simulations. Hence, these results indicate that α around 0.1 seems to be the best choice for the parameters that we have focused on in our simulation set up.

**Table 1:** **Results from the first simulation where three correlation settings (High, Mixture and Low) were considered, all with 25 significant predictor variables out of a total of 50,000 predictor variables**.

		**Lasso**	**EN075**	**EN05**	**EN04**	**EN03**	**EN02**	**EN015**	**EN01**	**EN005**	**EN001**
**minMSE**											
High LD	Correct	3 (0.99)	6 (1.69)	10 (2.36)	13 (2.42)	16 (2.43)	21 (1.99)	23 (1.89)	25 (0.66)	25 (0.00)	25 (0.00)
	False positive	5 (13.51)	5 (15.48)	8 (24.31)	8 (18.60)	7 (19.27)	10 (25.19)	10 (27.33)	15 (34.78)	35 (72.89)	689 (335)
	MSE	2.89 (0.11)	2.90 (0.11)	2.91 (0.11)	2.88 (0.10)	2.89 (0.11)	2.91 (0.11)	2.97 (0.08)	2.98 (0.08)	3.01 (0.08)	3.12 (0.09)
Mixed LD	Correct	3 (1.04)	5 (1.38)	9 (1.71)	11 (1.94)	14 (1.87)	17 (1.66)	18 (1.41)	20 (1.52)	24 (1.15)	25 (0.00)
	False positive	3 (23.47)	4 (16.78)	6 (19.96)	9 (21.46)	7 (20.20)	11 (32.30)	17 (30.23)	25 (43.00)	59 (87.53)	898 (369)
	MSE	2.92 (0.10)	2.93 (0.10)	2.94 (0.10)	2.94 (0.10)	2.96 (0.10)	2.97 (0.10)	2.98 (0.11)	2.99 (0.11)	3.02 (0.11)	3.16 (0.11)
Low LD	Correct	18 (1.87)	19 (1.88)	20 (1.78)	20 (1.44)	21 (1.33)	23 (1.23)	23 (0.91)	24 (0.66)	25 (0.24)	25 (0.00)
	False positive	7 (16.73)	8 (20.85)	10 (28.81)	9 (30.58)	10 (29.86)	13 (30.76)	27 (32.62)	30 (54.56)	73 (81.20)	1227 (406)
	MSE	3.10 (0.12)	3.10 (0.12)	3.09 (0.12)	3.08 (0.10)	3.09 (0.10)	3.09 (0.10)	3.10 (0.10)	3.11 (0.10)	3.14 (0.10)	3.31 (0.10)
**minMSE + 1SE**											
High LD	Correct	3 (0.91)	7 (1.80)	12 (2.38)	16 (2.32)	20 (2.05)	24 (1.22)	25 (0.81)	25 (0.10)	25 (0.00)	25 (0.00)
	False positive	0 (0.00)	0 (0.00)	0 (0.00)	0 (0.71)	0 (0.35)	0 (1.61)	0 (0.00)	0 (0.10)	0 (0.74)	35 (68)
	MSE	2.99 (0.12)	3.00 (0.11)	3.01 (0.12)	3.00 (0.12)	3.00 (0.12)	3.01 (0.10)	2.97 (0.08)	2.98 (0.09)	3.01 (0.08)	3.12 (0.10)
Mixed LD	Correct	3 (0.75)	6 (1.38)	10 (1.56)	12 (1.59)	14 (1.23)	16 (1.06)	17 (1.25)	18 (1.47)	24 (1.06)	25 (0.00)
	False positive	0 (0.00)	0 (0.00)	0 (0.00)	0 (0.31)	0 (0.14)	0 (0.74)	0 (0.72)	0 (1.19)	0 (2.24)	38 (64.72)
	MSE	3.03 (0.11)	3.04 (0.11)	3.06 (0.11)	3.06 (0.11)	3.06 (0.11)	3.08 (0.11)	3.11 (0.11)	3.10 (0.12)	3.13 (0.12)	3.26 (0.11)
Low LD	Correct	17 (2.12)	18 (1.89)	19 (1.88)	20 (1.49)	21 (1.36)	23 (1.22)	24 (0.78)	25 (0.51)	25 (0.00)	25 (0.00)
	False positive	0 (0.39)	0 (0.65)	0 (0.48)	0 (0.56)	0 (0.80)	0 (2.45)	0 (1.21)	0 (2.06)	0 (1.91)	106 (109)
	MSE	3.20 (0.13)	3.21 (0.13)	3.20 (0.13)	3.20 (0.12)	3.19 (0.11)	3.19 (0.12)	3.20 (0.11)	3.22 (0.10)	3.25 (0.10)	3.42 (0.11)

*Reported values are medians and SD (within parentheses) over 100 replicates*.

*The values of the elastic net (EN) refers to the penalty weight α*.

*The stopping criteria for λ were obtained with 10-fold cross-validation both at minimum MSE and minimum MSE plus 1 SE*.

*The median and standard deviation of MSE at the stopping criteria is also reported*.

### QTLMAS 2010 data

We used penalty weights of α = 1, 0.9, 0.75, 0.5, 0.3, 0.1, and 0.05. The average λ at minMSE + 1SE of ten 10-fold cross-validation runs at each α was considered to be optimal, to account for the minor differences in numbers of selected predictors between the repeats. Since the simulation procedure of this data makes it difficult to know the exact number of SNPs in LD with the 37 QTLs, we could not calculate the amount of correct or false positive predictor variables. However, we know that 364 SNPs were in moderate to high LD with the QTLs (*r*^2^ > 0.1), and that chromosome 5 has no QTLs.

**Figure 2:**
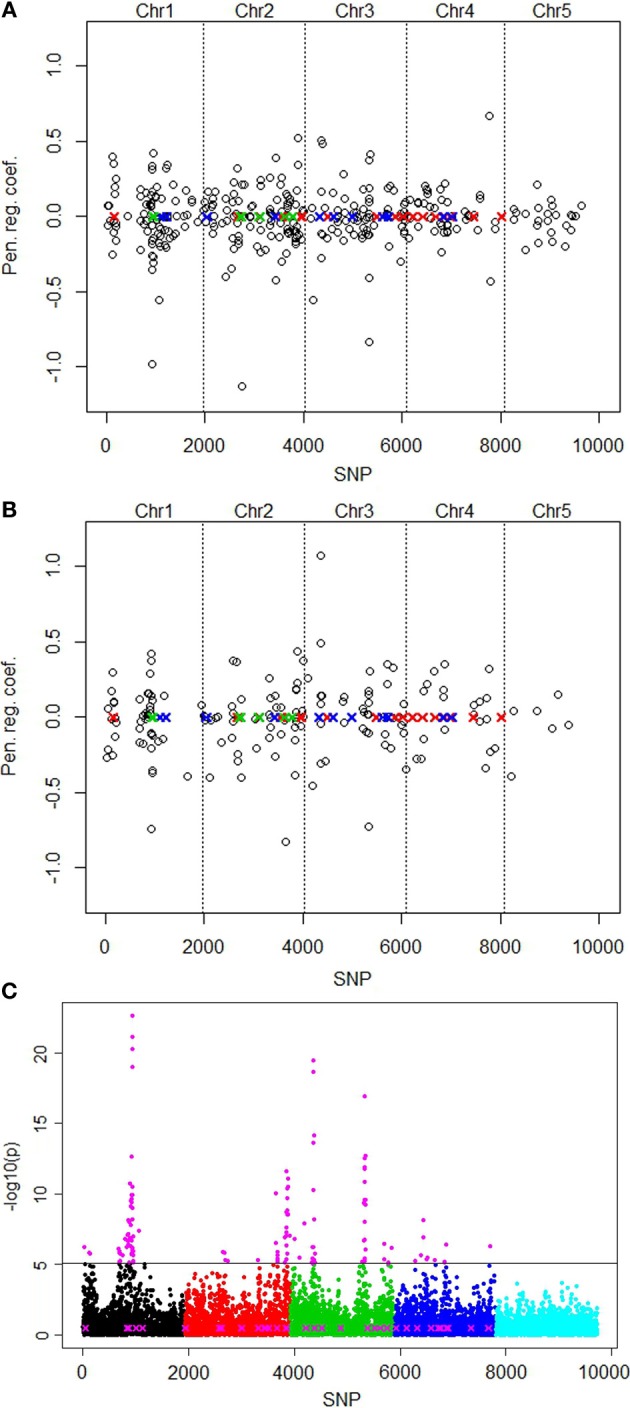
**Plots of positions and regression coefficients of the selected SNPs from the elastic net (EN01) analysis of the QTLMAS 2010 data in relation to the positions of the 37 simulated QTLs (X)**. The red, blue and green colors of the QTLs indicate additive positive, additive negative and epistatic (including imprinted) effects, respectively. The manhattan plot for the single marker regression shows the significance [-log10(*p*)-value] for all SNPs. **(A)** Without population structure correction. **(B)** With population structure correction using eigenvectors from spectral graph analysis. **(C)** Single marker regression with population structure correction. Highlighted markers (in magenta) are the important SNPs picked by the local false discovery rate method (Efron, 2010).

We first report the results without correction for population structure. The lasso picked out the smallest number of predictor variables (161) at minMSE + 1SE (Table 2). The elastic net with penalty weight α = 0.1 (EN01) selected 326 SNPs. These selected SNPs were located rather evenly over all 5 chromosomes, with the highest positive regression coefficient of SNP number 4609 on chromosome 3 (Figure 2A).

**Table 2:** **Results from the analysis of the simulated QTLMAS 2010 workshop data with and without correction for population structure (using eigenvectors from spectral graph analyses)**.

		**Lasso**	**EN09**	**EN075**	**EN05**	**EN03**	**EN01**	**EN005**	**fdr**
No pop. struct. corr.	Selected SNPs	161	176	168	219	232	326	454	78
	minMSE + 1SE	0.2825	0.3082	0.3822	0.5331	0.9087	2.6208	4.8283	–
Pop. struct. corr.	Selected SNPs	82	87	87	92	98	161	240	134
	minMSE + 1SE	0.2421	0.2594	0.3114	0.4673	0.7751	2.1707	4.0467	–

*The simulated pedigree consists of 3226 individuals from 5 generations*.

*The continuous trait was controlled by 37 QTLs that had 364 SNPs with *r*^2^ > 0.1. The stopping criteria for λ were obtained as the average of ten 10-fold cross-validation runs at minimum MSE plus 1 standard error*.

*The values of the elastic net (EN) refers to the penalty weight α (e.g., EN005 is elastic net with α = 0.05)*.

* fdr refers to the SNPs selected by the single marker regression local false discovery rate method (Efron, 2010)*.

The spectral graph method yielded 76 significant eigenvectors that we used to correct for population structure. The eigenvectors were set up as covariates that were forced to be in the model permanently without being penalized. With this approach, the lasso selected only 82 SNPs and EN01 161 SNPs (Table 2). The number of selected SNPs on chromosome 5 was reduced from 24 to 6 with the population structure correction for EN01 (Figure 2B). The single SNP regression results are shown in Figure 2C. For comparison purposes, the local false discovery rate was computed on the single marker regression *p*-values using the locfdr R package—134 influential SNPs were identified.

### Cattle data

In the Fleckvieh data set we used the same penalty weights as for the QTLMAS 2010 data, i.e., α = 1, 0.9, 0.75, 0.5, 0.3, 0.1, 0.05, and 0. Similarly to the QTLMAS2010 data set, ten 10-fold cross-validation runs at each α were used to find the average λ value pointing to minMSE + 1SE.

**Figure 3:**
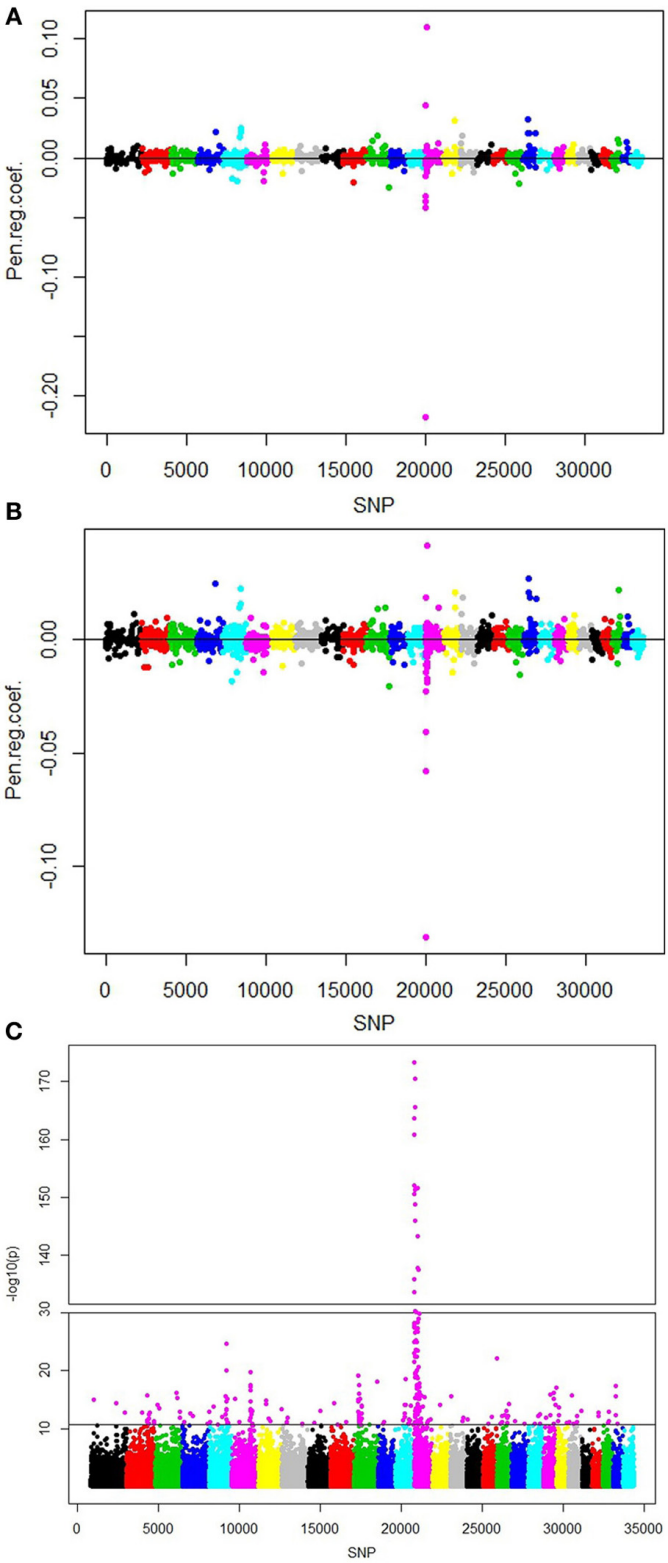
**Plots of positions and regression coefficients of the selected SNPs from (A) Lasso, (B) elastic net with α = 0.1, and (C) single marker regression with population structure correction for fat content in Fleckvieh bulls**. The manhattan plot shows the significance [-log10(*p*)-value] for all SNPs. Highlighted markers (in magenta) are the important SNPs picked by the local false discovery rate method (Efron, 2010).

The SNPs picked up by the various methods were between 1439 and 2689 for runs without population structure correction and between 1291 and 2504 for runs with correction for population structure (Table 3). The number of SNPs increased by a decreasing penalty factor in fairly regular manner when no population structure was applied, whereas the number of selected SNPs increased sharply from α = 0.3 to α = 0.1. We obtained 117 significant eigenvectors as a description of population structure in this data using the spectral graph method. With adjustment for population structure, the number of selected SNPs was lower in all cases. The selected SNPs were situated on all 29 autosomes, mostly with very low regression coefficients close to 0. All selected effects with corresponding regression coefficients from lasso and the elastic net with penalty weight α = 0.1 are shown in Figures 3A,B. The single SNP regression results for the cattle data set are shown in Figure 3C, with 160 influential SNPs identified by the local false discovery rate method.

**Table 3:** **Results from the analysis of the deregressed breeding value evaluation for fat content in Fleckvieh bulls**.

		**Lasso**	**EN09**	**EN075**	**EN05**	**EN03**	**EN01**	**EN005**	**fdr**
No pop. struct. corr.	Selected SNPs	1439	1451	1452	1556	1603	2142	2689	251
	minMSE + 1SE	0.0029	0.0033	0.0039	0.0057	0.0092	0.0240	0.0438	–
Pop. struct. corr.	Selected SNPs	1291	1291	1297	1400	1460	1966	2504	160
	minMSE + 1SE	0.0028	0.0031	0.0038	0.0055	0.0090	0.0236	0.0433	–

*34,373SNPs were analyzed on 5570 individuals with and without correction for population structure (using eigenvectors from spectral graph analyses)*.

*The stopping criteria for λ were obtained as the average of ten 10-fold cross-validation runs at minimum MSE plus 1 standard error*.

*The values of the elastic net refer to the penalty weight α (e.g., EN005 is elastic net with α = 0.05)*.

* fdr refers to the SNPs selected by the single marker regression local false discovery rate method (Efron, 2010)*.

The standard errors were similar between methods, but we noticed an increasing trend in standard error with decreasing penalty factor. When comparing the same methods with and without population structure correction, the standard error was similar for lasso and the elastic net with high penalty factors.

## Discussion

The goal of the first simulated data was to evaluate how different levels of correlation (linkage disequilibrium) between the predictor variables (SNP genotypes) influence the statistical properties of the lasso and the elastic net. Three correlation settings (high, mixed, and low LD) were considered, all with 25 influential predictor variables with known positions out of a total of 50,000 predictor variables. The response variable (phenotype) was chosen to eliminate complications in the variable selection that arise with high levels of error variance. When single SNP regression with a Bonferroni threshold was applied to the three LD scenarios it always resulted in 25 correctly selected variables and no false positives. However, it would be necessary to perform further simulation studies with other LD patterns before we can conclude that single SNP regression yields similar results as the elastic net.

The optimal value of the regularization parameter λ was obtained by performing 10-fold cross-validation to find the minMSE value and the minMSE + 1SE value. When minMSE was used as stopping criteria, the results show that an excessive amount of false positive predictor variables were selected under all three correlation settings. Moreover, the lasso and some elastic net procedures with high α values started to overfit without finding the correct number of true predictor variables. For the minMSE + 1SE stopping criterion, the lasso produced no false positives, but picked up too few correct SNP predictor variables for all three simulation settings. The elastic net generally performed better, with α = 0.1 resulting in no missing predictor variables and no false positives for the high and low correlation setting and only a few missing correct predictor variables in the mixed correlation setting. However, this α value never produced the lowest minMSE or minMSE + 1SE. Hence, the minimum of minMSE + 1SE should not be used to select variables over different α values. It should be noted that many factors can influence the tuning of λ and α. Waldron et al. (2011) used a 2D tuning approach for the elastic net penalties and found that a simultaneous tuning was required to differentiate it from the lasso and ridge regression. However, their approach is based on a combination of cross validation and quasi-Newton optimization to maximize the partial log-likelihood. It is unclear how our finding that the lowest minMSE doesn't correspond to the best penalty factors would influence their approach.

The purpose of the analyses of the second simulated data, from the QTLMAS 2010 workshop, was to evaluate the lasso and the elastic net methods on SNP and phenotype data that has been simulated under biologically more complex scenarios. Unfortunately, because of the complexity of the procedure generating this data, we have no exact number and positions of significantly associated SNPs. The continuous quantitative trait used in our study was determined by 37 QTLs, each QTL being surrounded by 19–47 polymorphic SNPs located within 1Mb distance from the QTL. 364 SNPs were found to be in moderate to high LD with the QTLs (*r*^2^ > 0.1). Hence, the true number of associated SNPs is likely be much larger than 37. Based on these figures, it seems as if the lasso once again selects too few predictor variables. The elastic net with α values around 0.1 appears to have superior performance. However, this conclusion should be interpreted with care since we don't know the exact number of associated SNPs. Recently, Mucha et al. (2011) published a compilation of results from the analyses of the QTLMAS 2010 data set. Seven different methods, including partial least-squares and Bayesian variable selection, were used by different research groups. None of the methods detected all of the 37 putative QTLs. The highest number of associated SNPs was detected by partial least-squares and BayesC. It is clear that these methods underestimate the number of SNPs that are in LD with the QTLs. Ogutu et al. (2012) applied the lasso, ridge regression and the elastic net to the QTLMAS 2011 data, but from a genomic selection perspective. Figure 2C shows the manhattan plot resulting from the single marker regression with population structure correction. The results are similar compared to the elastic net, with major peaks above the QTL positions. The fdr method selected 134 SNPs, similarly to the elastic net with alpha value 0.1 which identified 161 markers.

**Figure 4:**
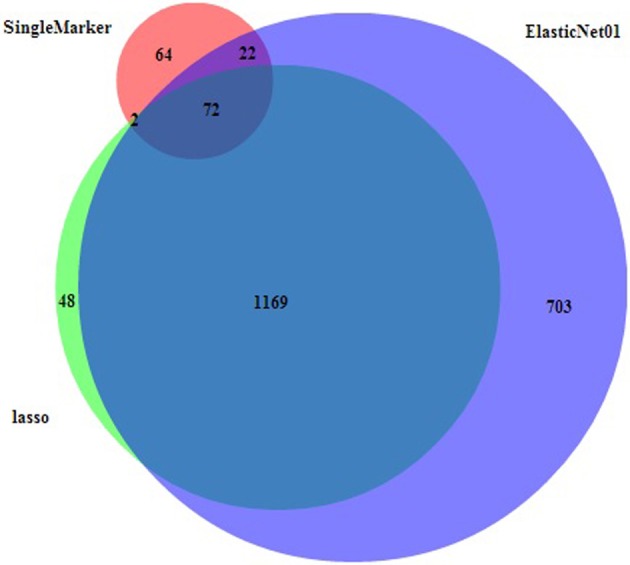
**Venn diagram (BioVenn, Hulsen et al., 2008) for number of overlapping markers selected by single marker regression (red), lasso (lime), and the elastic net (blue)**. The area of overlap is proportional to the number of commonly selected markers.

For the real cattle data the largest effects were on BTA5 (at 100.6 Mb), multiple SNPs on BTA14 (0.6–2.6 Mb), BTA15 (51.9 Mb) and BTA20 (27.2 Mb). Note that these conclusions are based on the elastic net with alpha value 0.1, but other alpha values tend to give similar results. The results were corresponding with those of Hayes et al. (2010) who found significant associations on BTA5, BTA14, and BTA20 in similar regions as in our study. Meredith et al. (2012) found moderate association on BTA15 in the 50 Mb region for protein content. The highest signal located on BTA14 points to the DGAT1, a gene with major effect on fat content (Grisart et al., 2002), located at 1.8 Mb. The single marker regression for the cattle data set resulted into 160 selected markers using the fdr methodology. This is a much lower number compared to the result from any of the penalized regression methodologies. The main peaks (Figure 3C) corresponded with the regions with the largest effects, identified by lasso and elastic net, with the majority of SNPs on the beginning of BTA14. Figure 4 shows the Venn diagram comparing the numbers of selected markers by single marker regression with the local false discovery rate method (Efron, 2010), lasso and elastic net with alpha value 0.1. The area circles relate to the number of selected markers, the area of overlap between the circles is proportional to the number of commonly selected markers using different methods. About one third of these were on BTA14, the rest in smaller groups on 16 other chromosomes. The list of the 72 SNPs selected by all three methods can be found in the supplement file 2. Almost all markers selected by lasso were also selected by the elastic net, which can be explained by their methodological similarities.

Although it is not expected that the single marker and penalized regression methods pick exactly the same SNPs, we note a relatively large difference in numbers of selected SNPs in the simulated and real data. We note that the deregressed breeding values that were used as phenotypes contain almost no error variance. This could be the reason for the large difference between the number of SNPs selected by single marker regression and the penalized regression methods. The −log10(*p*) values for the most significant region on BTA14 were unconventionally high as well, possibly for the same reason. When reducing the numbers of genotypes, the numbers of selected SNPs also decrease. One thousand nine hundred animals instead of the full set yielded a much lower number of SNPs (in the range of 500 based on the elastic net with alpha value 0.1). Moreover, when looking at the effect size of the selected SNPs, we see relatively high effect of each marker in the QTLMAS data set, but close to zero effects in the cattle data for the majority of the SNPs.

There are several applications of the lasso, ridge regression and elastic net to GWAS data (Malo et al., 2008; Wu et al., 2009; Cho et al., 2010). However, few studies have made statistical evaluations of these methods. Usai et al. (2009) tested the least angle regression version of the lasso on the QTLMAS 2008 data and found that 169 SNPs were needed to explain the variation of the 48 simulated QTLs. Yet, they used a rather ad hoc cross-validation approach where the highest correlation between genomic breeding values (sum of the regression coefficients of the SNPs) and true simulated breeding values was used as stopping criterion. This approach is difficult to generalize to real data because it relies on the fact that the breeding values are known or estimated without error. Ayers and Cordell (2010) compared the statistical properties of the lasso, ridge regression and elastic net on simulated data. However, the objectives of their study differ from our study. Firstly, Ayers and Cordell (2010) used a logistic model for binary phenotypes, whereas our study is focused on quantitative traits. Secondly, we have used cross-validation to obtain both minimum MSE and minimum MSE + 1SE, while Ayers and Cordell (2010) used a permutation approach aimed at controlling the Type I error rate. Thirdly, we have tried to argue that it is important to detect (map) all SNPs that have high effects on the phenotype, regardless if these are highly correlated with each other. Ayers and Cordell (2010) considered effects from groups of highly correlated variables as a single signal to prevent inflated false-positive rates, which we would regard more appropriate for prediction of future phenotypes.

It is well-known that population structure can lead to spurious false positive associations in GWA studies (Price et al., 2010; Sillanpää, 2011). There are several ways to adjust for population structure. If the pedigree is known, it is possible to use the additive relationship matrix as a structured random effect in a mixed model. Similarly, one can use the genomic relationship matrix based on identity-by-state coefficients. However, both these approaches are computationally demanding because of the need to solve large mixed model equations. Another approach is based on principal component analysis (PCA) where a certain number of important eigenvectors are calculated from a similarity matrix (estimated either based on the pedigree or the SNPs) between the individuals, and are used as covariates in the SNP regression model (Patterson et al., 2006). The computationally challenging part of this approach is the orthogonal decomposition of the similarity matrix. Lee et al. (2010) proposed spectral graph approach that in combination with simulation produces a certain number of significant eigenvectors that can be used for correction of population structure in GWAS. The correction was performed using the number of significant eigenvectors in our study, as we suspected some influence based on pedigree structure. It is important to fix the eigenvectors and not perform penalized variable selection on these because none of them were selected in our analyses if not treated as fixed. Further attempts to model population structure in a regularized regression framework include Puniyani et al. (2010).

Some recent studies have developed Bayesian versions of the elastic net. These methods are based on specifying shrinkage priors over the regression coefficients β. Bornn et al. (2010) suggested a hierarchical model with a prior distribution over β that combine and compromise between Laplace and Gaussian priors. This model requires computationally challenging tuning of two parameters that determines the amount of shrinkage. Li and Lin (2010) proposed a related Bayesian elastic net method with a slightly different specification of the prior where the two penalty parameters were chosen by the empirical Bayes method, whereas Kyung et al. (2010) showed that the two tuning parameters could be estimated within the Gibbs sampler by assigning hyperpriors to them. These methods are computationally demanding and it remains to be seen how large data they can handle can become. Simulation analyses in these two papers showed that the frequentist elastic net performed well in comparison with its Bayesian versions.

Finally, we would like to emphasize that the focus of this study has been on association analysis. GWAS can be interpreted as a mapping method where it is of importance to find all SNPs along the chromosomes that are associated with the phenotype. In statistical terms, GWAS should be based on the correct *estimation* of regression coefficients. On the other hand, in genome-wide selection (GS), the goal is to *predict* future observations based on a sub-set of SNPs that result in a high predictive accuracy. The number of SNPs and their chromosomal positions are of less concern in GS. It is difficult to draw any general conclusions about the predictive difference between the lasso and the elastic net based on our study. Further studies on the relation between estimation and prediction in highly multicollinear data and new criteria for variable selection and predictive performance are needed.

## Supplementary material

The Supplementary Material for this article can be found online at: http://www.frontiersin.org/journal/10.3389/fgene.2013.00270/abstract

### Conflict of interest statement

The authors declare that the research was conducted in the absence of any commercial or financial relationships that could be construed as a potential conflict of interest.

## References

[B1] Ayers K. L. Cordell H. J. (2010). SNP selection in genome-wide and candidate gene studies via penalized logistic regression. Genet. Epidemiol. 34, 879–891 10.1002/gepi.20543 21104890 PMC3410531

[B2] Bornn L. Gottardo R. Doucet A. (2010). Grouping priors and the Bayesian elastic net. Technical report 254, Department of Statistics. University of British Columbia arXiv:1001.4083.

[B3] Bühlmann P. van de Geer S. (2011). Statistics for High-Dimensional Data: Methods, Theory and Applications. New York, NY: Springer-Verlag 10.1007/978-3-642-20192-9

[B4] Cantor R. M. Lange K. Sinsheimer J. S. (2010). Prioritizing GWAS results: a review of statistical methods and recommendations for their application. Am. J. Hum. Genet. 86, 6–22 10.1016/j.ajhg.2009.11.017 20074509 PMC2801749

[B5] Cho S. Kim K. Kim Y. J. Lee J. K. Cho Y. S. Lee J. Y. (2010). Joint identification of multiple genetic variants via elastic-net variable selection in a genome-wide association analysis. Ann. Hum. Genet. 74, 416–428 10.1111/j.1469-1809.2010.00597.x 20642809

[B6] Dasgupta A. Sun Y. V. König I. R. Bailey-Wilson J. E. Malley J. D. (2011). Brief review of regression-based and machine learning methods in genetic epidemiology: the Genetic Analysis Workshop 17 experience. Genet. Epidemiol. 35(Suppl. 1), S5–S11 10.1002/gepi.20642 22128059 PMC3345521

[B7] de Los Campos G. Hickey J. M. Pong-Wong R. Daetwyler H. D. Calus M. P. (2013). Whole-genome regression and prediction methods applied to plant and animal breeding. Genetics 193, 327–345 10.1534/genetics.112.143313 22745228 PMC3567727

[B8] Efron B. (2010). Large-Scale Inference: Empirical Bayes Methods for Estimation, Testing, and Prediction. Cambrige, UK: Cambrige University Press 10.1017/CBO9780511761362

[B9] Efron B. Turnbull B. B. Narasimhan B. (2011). Locfdr: Computes Local False Discovery Rates. R package Version 1.1-7. Available online at: http://CRAN.R-project.org/package=locfdr

[B10] Fadista J. Bendixen C. (2012). Genomic position mapping discrepancies of commercial SNP chips. PLoS ONE 7:e31025 10.1371/journal.pone.0031025 22363540 PMC3281913

[B11] Fan J. Lv J. (2010). A selective overview of variable selection in high dimensional feature space. Stat. Sin. 20, 101–148 21572976 PMC3092303

[B12] Friedman J. Hastie T. Hoefling H. Tibshirani R. (2007). Pathwise coordinate optimization. Ann. Appl. Stat. 2, 302–332 10.1214/07-AOAS131

[B13] Friedman J. Hastie T. Tibshirani R. (2010). Regularization paths for generalized linear models via coordinate descent. J. Stat. Softw. 33, 1–22 20808728 PMC2929880

[B14] Garrick D. J. Taylor J. F. Fernando R. L. (2009). Deregressing estimated breeding values and weighting information for genomic regression analyses. Genet. Sel. Evol. 41, 55 10.1186/1297-9686-41-55 20043827 PMC2817680

[B15] Gianola G. (2013). Priors in whole-genome regression: the Bayesian alphabet returns. Genetics 194, 573–596 10.1534/genetics.113.151753 23636739 PMC3697965

[B16] Goddard M. E. Hayes B. J. (2009). Mapping genes for complex traits in domestic animals and their use in breeding programmes. Nat. Rev. Genet. 10, 381–391 10.1038/nrg2575 19448663

[B17] Grisart B. Coppieters W. Farnir F. Karim L. Ford C. Berzi P. (2002). Positional candidate cloning of a QTL in dairy cattle: identification of a missense mutation in the bovine DGAT1 gene with major effect on milk yield and composition. Genome Res. 12, 222–231 10.1101/gr.224202 11827942

[B18] Hans C. (2010). Model uncertainty and variable selection in Bayesian lasso regression. Stat. Comput. 20, 221–229 10.1007/s11222-009-9160-922549815

[B19] Hastie T. R. Tibshirani R. Friedman J. (2009). Elements of Statistical Learning: Data Mining, Inference and Prediction, 2nd Edn. New York, NY: Springer-Verlag

[B20] Hayes B. J. Pryce J. Chamberlain A. J. Bowman P. J. Goddard M. E. (2010). Genetic architecture of complex traits and accuracy of genomic prediction: coat colour, milk-fat percentage, and type in Holstein cattle as contrasting model traits. PLoS Genet. 6:e1001139 10.1371/journal.pgen.1001139 20927186 PMC2944788

[B21] Heffner E. L. Sorrels M. E. Jannink J. L. (2009). Genomic selection for crop improvement. Crop. Sci. 49, 1–12 10.2135/cropsci2008.08.0512

[B22] Hesterberg T. Choi N. H. Meier L. Fraley C. (2008). Least angle and ℓ_1_ penalized regression: a review. Stat. Surv. 2, 61–93 10.1214/08-SS035

[B23] Hoerl A. E. Kennard R. (1970). Ridge regression: biased estimation for non orthogonal problems. Technometrics 12, 55–67 10.1080/00401706.1970.10488634

[B24] Hulsen T. de Vlieg J. Alkema W. (2008). BioVenn - a web application for the comparison and visualization of biological lists using area-proportional Venn diagrams. BMC Genomics 9:488 10.1186/1471-2164-9-488 18925949 PMC2584113

[B25] Klei L. Kent B. P. Melhem N. Devlin B. Roeder K. (2011). GemTools: a Fast and Efficient Approach to Estimating Genetic Ancestry. Available online at: http://arxiv.org/pdf/1104.1162

[B26] Kooperberg C. LeBlanc M. Obenchain V. (2010). Risk prediction using genome-wide association studies. Genet. Epidemiol. 34, 643–652 10.1002/gepi.20509 20842684 PMC2964405

[B27] Kyung M. Gill J. Ghosh M. Casella G. (2010). Penalized regression, standard errors, and Bayesian lassos. Bay. Anal. 5, 369–412 10.1214/10-BA607

[B28] Lee A. B. Luca D. Roeder K. (2010). A spectral graph approach to discovering genetic ancestry. Ann. Appl. Stat. 4, 179–202 10.1214/09-AOAS281 20689656 PMC2916191

[B29] Li Q. Lin N. (2010). The Bayesian elastic net. Bay. Anal. 5, 151–170 10.1214/10-BA506

[B30] Malo N. Libiger O. Schork N. J. (2008). Accommodating linkage disequilibrium in genetic-association analyses via ridge regression. Am. J. Hum. Genet. 82, 375–385 10.1016/j.ajhg.2007.10.012 18252218 PMC2427310

[B31] McCarthy M. I. Abecasis G. R. Cardon L. R. Goldstein D. B. Little J. Ioannidis J. P. A. (2008). Genome-wide association studies for complex traits: consensus,uncertainty and challenges. Nat. Rev. Genet. 9, 356–369 10.1038/nrg2344 18398418

[B32] Meredith B. K. Kearney F. J. Finlay E. K. Bradley D. G. Fahey A. G. Berry D. P. (2012). Genome-wide associations for milk production and somatic cell score in Holstein-Friesian cattle in Ireland. BMC Genet. 13:21 10.1186/1471-2156-13-21 22449276 PMC3361482

[B33] Moore J. H. Asselbergs F. W. Williams S. M. (2010). Bioinformatics challenges for genome-wide association studies. Bioinformatics, 26, 445–455 10.1093/bioinformatics/btp713 20053841 PMC2820680

[B34] Mucha S. Pszczoła M. Strabel T. Wolc A. Paczyñska P. Szydlowski M. (2011). Comparison of analyses of the QTLMAS XIV common dataset. II: QTL analysis. BMC Proc. 5(Suppl 3):S2 10.1186/1753-6561-5-S3-S2 21624172 PMC3103201

[B35] Ogutu J. O. Schulz-Streeck T. Piepho H. P. (2012). Genomic selection using regularized linear regression models: ridge regression, lasso, elastic net and their extensions. BMC Proc. 6(Suppl. 2):S10 10.1186/1753-6561-6-S2-S10 22640436 PMC3363152

[B36] Patterson N. Price A. L. Reich D. (2006). Population structure and eigenanalysis. PLoS Genet. 2:e190 10.1371/journal.pgen.0020190 17194218 PMC1713260

[B37] Price A. L. Zaitlen N. A. Reich D. Patterson N. (2010). New approaches to population stratification in genome-wide association studies. Nat. Rev. Genet. 11, 459–463 10.1038/nrg2813 20548291 PMC2975875

[B38] Puniyani K. Kim S. Xing E. P. (2010). Multi-population GWA mapping via multi-task regularized regression. Bioinformatics 26, 208–216 10.1093/bioinformatics/btq191 20529908 PMC2881376

[B39] Rice T. K. Schork N. J. Rao D. C. (2008). Methods for handling multiple testing. Adv. Genet. 60, 293–308 10.1016/S0065-2660(07)00412-9 18358325

[B40] Sillanpää M. J. (2011). Overview of techniques to account for confounding due to population stratification and cryptic relatedness in genomic data association analyses. Heredity 106, 511–519 10.1038/hdy.2010.91 20628415 PMC3183892

[B41] Sung Y. J. Rice T. K. Shi G. Gu C. C. Rao D. C. (2009). Comparison between single-marker analysis using Merlin and multi-marker analysis using LASSO for Framingham simulated data. BMC Proc. 3(Suppl. 7):S27 10.1186/1753-6561-3-s7-s27 20018017 PMC2795924

[B42] Szydłowski M. Paczyñska P. (2011). QTLMAS 2010: simulated dataset. BMC Proc. 5(Suppl. 3):S3 10.1186/1753-6561-5-S3-S3 21624173 PMC3103202

[B43] Szymczak S. Biernacka J. M. Cordell H. J. González-Recio O. König I. R. Zhang H. (2009). Machine learning in genome-wide association studies. Genet. Epidemiol. 33, S51–S57 10.1002/gepi.20473 19924717

[B44] Tibshirani R. (1996). Regression shrinkage and selection via the lasso. J. Roy. Stat. Soc. B 58, 267–288 10.1111/j.1467-9868.2011.00771.x

[B45] Tutz G. Ulbricht J. (2009). Penalized regression with correlation-based penalty. Stat. Comp. 19, 239–253 10.1007/s11222-008-9088-5

[B46] Usai M. G. Goddard M. E. Hayes B. J. (2009). LASSO with cross-validation for genomic selection. Genet. Res. 91, 427–436 10.1017/S0016672309990334 20122298

[B47] Waldron L. Pintilie M. Tsao M. S. Shepherd F. A. Huttenhower C. Jurisica I. (2011). Optimized application of penalized regression methods to diverse genomic data. Bioinformatics 27, 3399–3406 10.1093/bioinformatics/btr591 22156367 PMC3232376

[B48] Wu T. T. Chen Y. F. Hastie T. Sobel E. Lange K. (2009). Genome-wide association analysis by lasso penalized logistic regression. Bioinformatics 25, 714–721 10.1093/bioinformatics/btp041 19176549 PMC2732298

[B49] Zou H. Hastie T. (2005). Regularization and variable selection via the elastic net. J. Roy. Stat. Soc. B 67, 301–320 10.1111/j.1467-9868.2005.00503.x

