# Corrigendum: Evaluation of the lasso and the elastic net in genome-wide association studies

**DOI:** 10.3389/fgene.2014.00349

**Published:** 2014-10-01

**Authors:** Patrik Waldmann, Gábor Mészáros, Birgit Gredler, Christian Fuerst, Johann Sölkner

**Affiliations:** ^1^Division of Livestock Sciences, Department of Sustainable Agricultural Systems, University of Natural Resources and Life SciencesVienna, Austria; ^2^Division of Statistics, Department of Computer and Information Science, Linköping UniversityLinköping, Sweden; ^3^Qualitas AGZug, Switzerland; ^4^ZuchtData EDV-Dienstleistungen GmbHVienna, Austria

**Keywords:** LASSO, elastic net, simulation, GWAS, population structure, cattle

Translational research program is intended to provide scientists with an incentive to examine research findings from the perspective of concrete applications or other uses, and to give outstanding researchers an opportunity to develop these findings into specific applications and/or economic, societal or social benefits. This program is jointly funded by the Austrian Science Fund (FWF) and the Austrian Ministry for Transport, Innovation and Technology (BMVIT). Therefore, the acknowledgments section of the paper “Evaluation of the lasso and the elastic net in genome-wide association studies” should state:

“The financial support of the Austrian Ministry for Transport, Innovation and Technology (BMVIT) and the Austrian Science Fund (FWF) via the project TRP46-B19 is greatly acknowledged.”

## Conflict of interest statement

The authors declare that the research was conducted in the absence of any commercial or financial relationships that could be construed as a potential conflict of interest.

