# Supplementary material for: Evaluation of the lasso and the elastic net in genome-wide association studies
Source: Front Genet. 2013 Dec 4;4:270. doi: 10.3389/fgene.2013.00270 (PMC3850240; doi:10.3389/fgene.2013.00270)
Supplement: Supplementary file 1 [file DataSheet1.PDF]

```
########################################################
# Low LD setting
########################################################
rm(list=ls(all=TRUE))

library(Matrix)
library(glmnet)

resmat1se<-matrix(NA,nrow=100,ncol=20)
resmatmin<-matrix(NA,nrow=100,ncol=20)
resmat<-matrix(NA,nrow=100,ncol=20)

# 100 replicates of the LowLD setting
for(i in 1:100){

# generation of simulated data
ylow=rnorm(500,-5,1)
yhigh=rnorm(500,5,1)
ytot=c(ylow,yhigh) # phenotype


x1=Matrix(rbinom(5e+07,1,0.5),nrow=1000,ncol=50000)
x2=Matrix(rbinom(5e+07,1,0.5),nrow=1000,ncol=50000)

x<- x1+x2 # genotypes with 0-1-2 coding


x[,990] = sort(x[,990])
indind<-1:1000
sampind<-sample(indind,250)
xsel<-x[sampind,990]
xperm<-sample(xsel)
x[sampind,990]<-xperm
x[,995] = sort(x[,995])
sampind<-sample(indind,250)
xsel<-x[sampind,995]
xperm<-sample(xsel)
x[sampind,995]<-xperm
x[,1000] = sort(x[,1000])
sampind<-sample(indind,250)
xsel<-x[sampind,1000]
xperm<-sample(xsel)
x[sampind,1000]<-xperm
x[,1005] = sort(x[,1005])
sampind<-sample(indind,250)
xsel<-x[sampind,1005]
xperm<-sample(xsel)
x[sampind,1005]<-xperm
x[,1010] = sort(x[,1010])
sampind<-sample(indind,250)
xsel<-x[sampind,1010]
xperm<-sample(xsel)
x[sampind,1010]<-xperm
```

```
x[,9990] = sort(x[,9990])
sampind<-sample(indind,250)
xsel<-x[sampind,9990]
xperm<-sample(xsel)
x[sampind,9990]<-xperm
x[,9995] = sort(x[,9995])
sampind<-sample(indind,250)
xsel<-x[sampind,9995]
xperm<-sample(xsel)
x[sampind,9995]<-xperm
x[,10000] = sort(x[,10000])
sampind<-sample(indind,250)
xsel<-x[sampind,10000]
xperm<-sample(xsel)
x[sampind,10000]<-xperm
x[,10005] = sort(x[,10005])
sampind<-sample(indind,250)
xsel<-x[sampind,10005]
xperm<-sample(xsel)
x[sampind,10005]<-xperm
x[,10010] = sort(x[,10010])
sampind<-sample(indind,250)
xsel<-x[sampind,10010]
xperm<-sample(xsel)
x[sampind,10010]<-xperm

x[,19990] = sort(x[,19990])
sampind<-sample(indind,250)
xsel<-x[sampind,19990]
xperm<-sample(xsel)
x[sampind,19990]<-xperm
x[,19995] = sort(x[,19995])
sampind<-sample(indind,250)
xsel<-x[sampind,19995]
xperm<-sample(xsel)
x[sampind,19995]<-xperm
x[,20000] = sort(x[,20000])
sampind<-sample(indind,250)
xsel<-x[sampind,20000]
xperm<-sample(xsel)
x[sampind,20000]<-xperm
x[,20005] = sort(x[,20005])
sampind<-sample(indind,250)
xsel<-x[sampind,20005]
xperm<-sample(xsel)
x[sampind,20005]<-xperm
x[,20010] = sort(x[,20010])
sampind<-sample(indind,250)
xsel<-x[sampind,20010]
xperm<-sample(xsel)
x[sampind,20010]<-xperm
```

```
x[,29990] = sort(x[,29990])
sampind<-sample(indind,250)
xsel<-x[sampind,29990]
xperm<-sample(xsel)
x[sampind,29990]<-xperm
x[,29995] = sort(x[,29995])
sampind<-sample(indind,250)
xsel<-x[sampind,29995]
xperm<-sample(xsel)
x[sampind,29995]<-xperm
x[,30000] = sort(x[,30000])
sampind<-sample(indind,250)
xsel<-x[sampind,30000]
xperm<-sample(xsel)
x[sampind,30000]<-xperm
x[,30005] = sort(x[,30005])
sampind<-sample(indind,250)
xsel<-x[sampind,30005]
xperm<-sample(xsel)
x[sampind,30005]<-xperm
x[,30010] = sort(x[,30010])
sampind<-sample(indind,250)
xsel<-x[sampind,30010]
xperm<-sample(xsel)
x[sampind,30010]<-xperm

x[,39990] = sort(x[,39990])
sampind<-sample(indind,250)
xsel<-x[sampind,39990]
xperm<-sample(xsel)
x[sampind,39990]<-xperm
x[,39995] = sort(x[,39995])
sampind<-sample(indind,250)
xsel<-x[sampind,39995]
xperm<-sample(xsel)
x[sampind,39995]<-xperm
x[,40000] = sort(x[,40000])
sampind<-sample(indind,250)
xsel<-x[sampind,40000]
xperm<-sample(xsel)
x[sampind,40000]<-xperm
x[,40005] = sort(x[,40005])
sampind<-sample(indind,250)
xsel<-x[sampind,40005]
xperm<-sample(xsel)
x[sampind,40005]<-xperm
x[,40010] = sort(x[,40010])
sampind<-sample(indind,250)
xsel<-x[sampind,40010]
xperm<-sample(xsel)
x[sampind,40010]<-xperm

true<-c(990,995,1000,1005,1010,
```

```
9990,9995,10000,10005,10010,
19990,19995,20000,20005,20010,
29990,29995,30000,30005,30010,
39990,39995,40000,40005,40010)

##########
# Lasso and elastic net analyses with cross validation
##########

#Lasso
cvmseLasso=cv.glmnet(x,ytot)
resmat[i,1]<-min(cvmseLasso$cvm)
resmat[i,2]<-cvmseLasso$cvm[which(cvmseLasso$lambda==cvmseLasso$lambda.1se)]

Lassopick<-which(coef(cvmseLasso,s="lambda.1se")>0)-1
Lassopick<-Lassopick[which(Lassopick>0)]
Lassotrue<-intersect(Lassopick,true)
nLassotrue<-length(Lassotrue)
nLassofalse<-length(Lassopick)-length(Lassotrue)


Lassopickmin<-which(coef(cvmseLasso,s="lambda.min")>0)-1
Lassopickmin<-Lassopickmin[which(Lassopickmin>0)]
Lassotruemin<-intersect(Lassopickmin,true)
nLassotruemin<-length(Lassotruemin)
nLassofalsemin<-length(Lassopickmin)-length(Lassotruemin)

# Elastic net – alpha 0.75
cvmseEN075=cv.glmnet(x,ytot,alpha=0.75)
resmat[i,3]<-min(cvmseEN075$cvm)
resmat[i,4]<-cvmseEN075$cvm[which(cvmseEN075$lambda==cvmseEN075$lambda.1se)]

EN075pick<-which(coef(cvmseEN075,s="lambda.1se")>0)-1
EN075pick<-EN075pick[which(EN075pick>0)]
EN075true<-intersect(EN075pick,true)
nEN075true<-length(EN075true)
nEN075false<-length(EN075pick)-length(EN075true)

EN075pickmin<-which(coef(cvmseEN075,s="lambda.min")>0)-1
EN075pickmin<-EN075pickmin[which(EN075pickmin>0)]
EN075truemin<-intersect(EN075pickmin,true)
nEN075truemin<-length(EN075truemin)
nEN075falsemin<-length(EN075pickmin)-length(EN075truemin)

# Elastic net – alpha 0.5
cvmseEN05=cv.glmnet(x,ytot,alpha=0.5)
resmat[i,5]<-min(cvmseEN05$cvm)
resmat[i,6]<-cvmseEN05$cvm[which(cvmseEN05$lambda==cvmseEN05$lambda.1se)]

EN05pick<-which(coef(cvmseEN05,s="lambda.1se")>0)-1
EN05pick<-EN05pick[which(EN05pick>0)]
EN05true<-intersect(EN05pick,true)
nEN05true<-length(EN05true)
```

```
nEN05false<-length(EN05pick)-length(EN05true)

EN05pickmin<-which(coef(cvmseEN05,s="lambda.min")>0)-1
EN05pickmin<-EN05pickmin[which(EN05pickmin>0)]
EN05truemin<-intersect(EN05pickmin,true)
nEN05truemin<-length(EN05truemin)
nEN05falsemin<-length(EN05pickmin)-length(EN05truemin)

# Elastic net – alpha 0.4
cvmseEN04<-cv.glmnet(x,ytot,alpha=0.4)
resmat[i,7]<-min(cvmseEN04$cvm)
resmat[i,8]<-cvmseEN04$nzero[which(cvmseEN04$lambda==cvmseEN04$lambda.1se)]

EN04pick<-which(coef(cvmseEN04,s="lambda.1se")>0)-1
EN04pick<-EN04pick[which(EN04pick>0)]
EN04true<-intersect(EN04pick,true)
nEN04true<-length(EN04true)
nEN04false<-length(EN04pick)-length(EN04true)

EN04pickmin<-which(coef(cvmseEN04,s="lambda.min")>0)-1
EN04pickmin<-EN04pickmin[which(EN04pickmin>0)]
EN04truemin<-intersect(EN04pickmin,true)
nEN04truemin<-length(EN04truemin)
nEN04falsemin<-length(EN04pickmin)-length(EN04truemin)

# Elastic net – alpha 0.3
cvmseEN03=cv.glmnet(x,ytot,alpha=0.3)
resmat[i,9]<-min(cvmseEN03$cvm)
resmat[i,10]<-cvmseEN03$cvm[which(cvmseEN03$lambda==cvmseEN03$lambda.1se)]

EN03pick<-which(coef(cvmseEN03,s="lambda.1se")>0)-1
EN03pick<-EN03pick[which(EN03pick>0)]
EN03true<-intersect(EN03pick,true)
nEN03true<-length(EN03true)
nEN03false<-length(EN03pick)-length(EN03true)

EN03pickmin<-which(coef(cvmseEN03,s="lambda.min")>0)-1
EN03pickmin<-EN03pickmin[which(EN03pickmin>0)]
EN03truemin<-intersect(EN03pickmin,true)
nEN03truemin<-length(EN03truemin)
nEN03falsemin<-length(EN03pickmin)-length(EN03truemin)

# Elastic net – alpha 0.2
cvmseEN02=cv.glmnet(x,ytot,alpha=0.2)
resmat[i,11]<-min(cvmseEN02$cvm)
resmat[i,12]<-cvmseEN02$nzero[which(cvmseEN02$lambda==cvmseEN02$lambda.1se)]

EN02pick<-which(coef(cvmseEN02,s="lambda.1se")>0)-1
EN02pick<-EN02pick[which(EN02pick>0)]
EN02true<-intersect(EN02pick,true)
nEN02true<-length(EN02true)
nEN02false<-length(EN02pick)-length(EN02true)
```

```
EN02pickmin<-which(coef(cvmseEN02,s="lambda.min")>0)-1
EN02pickmin<-EN02pickmin[which(EN02pickmin>0)]
EN02truemin<-intersect(EN02pickmin,true)
nEN02truemin<-length(EN02truemin)
nEN02falsemin<-length(EN02pickmin)-length(EN02truemin)

# Elastic net – alpha 0.15
cvmseEN015=cv.glmnet(x,ytot,alpha=0.15)
resmat[i,13]<-min(cvmseEN015$cvm)
resmat[i,14]<-cvmseEN015$nzero[which(cvmseEN015$lambda==cvmseEN015$lambda.1se)]

EN015pick<-which(coef(cvmseEN015,s="lambda.1se")>0)-1
EN015pick<-EN015pick[which(EN015pick>0)]
EN015true<-intersect(EN015pick,true)
nEN015true<-length(EN015true)
nEN015false<-length(EN015pick)-length(EN015true)

EN015pickmin<-which(coef(cvmseEN015,s="lambda.min")>0)-1
EN015pickmin<-EN015pickmin[which(EN015pickmin>0)]
EN015truemin<-intersect(EN015pickmin,true)
nEN015truemin<-length(EN015truemin)
nEN015falsemin<-length(EN015pickmin)-length(EN015truemin)

# Elastic net – alpha 0.1
cvmseEN01=cv.glmnet(x,ytot,alpha=0.1)
resmat[i,15]<-min(cvmseEN01$cvm)
resmat[i,16]<-cvmseEN01$cvm[which(cvmseEN01$lambda==cvmseEN01$lambda.1se)]

EN01pick<-which(coef(cvmseEN01,s="lambda.1se")>0)-1
EN01pick<-EN01pick[which(EN01pick>0)]
EN01true<-intersect(EN01pick,true)
nEN01true<-length(EN01true)
nEN01false<-length(EN01pick)-length(EN01true)

EN01pickmin<-which(coef(cvmseEN01,s="lambda.min")>0)-1
EN01pickmin<-EN01pickmin[which(EN01pickmin>0)]
EN01truemin<-intersect(EN01pickmin,true)
nEN01truemin<-length(EN01truemin)
nEN01falsemin<-length(EN01pickmin)-length(EN01truemin)

# Elastic net – alpha 0.05
cvmseEN005=cv.glmnet(x,ytot,alpha=0.05)
resmat[i,17]<-min(cvmseEN005$cvm)
resmat[i,18]<-cvmseEN005$cvm[which(cvmseEN005$lambda==cvmseEN005$lambda.1se)]

EN005pick<-which(coef(cvmseEN005,s="lambda.1se")>0)-1
EN005pick<-EN005pick[which(EN005pick>0)]
EN005true<-intersect(EN005pick,true)
nEN005true<-length(EN005true)
nEN005false<-length(EN005pick)-length(EN005true)

EN005pickmin<-which(coef(cvmseEN005,s="lambda.min")>0)-1
EN005pickmin<-EN005pickmin[which(EN005pickmin>0)]
```

```
EN005truemin<-intersect(EN005pickmin,true)
nEN005truemin<-length(EN005truemin)
nEN005falsemin<-length(EN005pickmin)-length(EN005truemin)

# Elastic net – alpha 0.01
cvmseEN001=cv.glmnet(x,ytot,alpha=0.01)
resmat[i,19]<-min(cvmseEN001$cvm)
resmat[i,20]<-cvmseEN001$cvm[which(cvmseEN001$lambda==cvmseEN001$lambda.1se)]

EN001pick<-which(coef(cvmseEN001,s="lambda.1se")>0)-1
EN001pick<-EN001pick[which(EN001pick>0)]
EN001true<-intersect(EN001pick,true)
nEN001true<-length(EN001true)
nEN001false<-length(EN001pick)-length(EN001true)

EN001pickmin<-which(coef(cvmseEN001,s="lambda.min")>0)-1
EN001pickmin<-EN001pickmin[which(EN001pickmin>0)]
EN001truemin<-intersect(EN001pickmin,true)
nEN001truemin<-length(EN001truemin)
nEN001falsemin<-length(EN001pickmin)-length(EN001truemin)

resmat1se[i,]<-c(nLassotrue,nLassofalse,nEN075true,nEN075false,nEN05true,nEN05false,
                 nEN04true,nEN04false,nEN03true,nEN03false,nEN02true,nEN02false,
                 nEN015true,nEN015false,nEN01true,nEN01false,nEN005true,nEN005false,
                 nEN001true,nEN001false)
resmatmin[i,]<-c(nLassotruemin,nLassofalsemin,nEN075truemin,nEN075falsemin,
                  nEN05truemin,nEN05falsemin,nEN04truemin,nEN04falsemin,
                  nEN03truemin,nEN03falsemin,nEN02truemin,nEN02falsemin,
                  nEN015truemin,nEN015falsemin,nEN01truemin,nEN01falsemin,
                  nEN005truemin,nEN005falsemin,nEN001truemin,nEN001falsemin)
}

dput(resmat,"SimResLowLDMSE.txt")
dput(resmat1se," SimResLowLD1se.txt")
dput(resmatmin," SimResLowLDmin.txt")

########################################################
# Mixed LD setting
########################################################
rm(list=ls(all=TRUE))

library(Matrix)
library(glmnet)

resmat1se<-matrix(NA,nrow=100,ncol=20)
resmatmin<-matrix(NA,nrow=100,ncol=20)
resmat<-matrix(NA,nrow=100,ncol=20)

# 100 replicates of the MixedLD setting
for(i in 1:100){

# generation of simulated data
ylow=rnorm(500,-5,1)
```

```
yhigh=rnorm(500,5,1)
ytot=c(ylow,yhigh) # phenotype

x1=Matrix(rbinom(5e+07,1,0.5),nrow=1000,ncol=50000)
x2=Matrix(rbinom(5e+07,1,0.5),nrow=1000,ncol=50000)

x<- x1+x2 # genotypes with 0-1-2 coding


x[,990] = sort(x[,990])
indind<-1:1000
sampind<-sample(indind,250)
xsel<-x[sampind,990]
xperm<-sample(xsel)
x[sampind,990]<-xperm
x[,995] = sort(x[,995])
sampind<-sample(indind,250)
xsel<-x[sampind,995]
xperm<-sample(xsel)
x[sampind,995]<-xperm
x[,1000] = sort(x[,1000])
sampind<-sample(indind,250)
xsel<-x[sampind,1000]
xperm<-sample(xsel)
x[sampind,1000]<-xperm
x[,1005] = sort(x[,1005])
sampind<-sample(indind,250)
xsel<-x[sampind,1005]
xperm<-sample(xsel)
x[sampind,1005]<-xperm
x[,1010] = sort(x[,1010])
sampind<-sample(indind,250)
xsel<-x[sampind,1010]
xperm<-sample(xsel)
x[sampind,1010]<-xperm

x[,9990] = sort(x[,9990])
x[,9995] = sort(x[,9995])
x[,10000] = sort(x[,10000])
x[,10005] = sort(x[,10005])
x[,10010] = sort(x[,10010])

x[,19990] = sort(x[,19990])
x[,19995] = sort(x[,19995])
x[,20000] = sort(x[,20000])
x[,20005] = sort(x[,20005])
x[,20010] = sort(x[,20010])

x[,29990] = sort(x[,29990])
x[,29995] = sort(x[,29995])
x[,30000] = sort(x[,30000])
x[,30005] = sort(x[,30005])
x[,30010] = sort(x[,30010])
```

```
x[,39990] = sort(x[,39990])
sampind<-sample(indind,250)
xsel<-x[sampind,39990]
xperm<-sample(xsel)
x[sampind,39990]<-xperm
x[,39995] = sort(x[,39995])
sampind<-sample(indind,250)
xsel<-x[sampind,39995]
xperm<-sample(xsel)
x[sampind,39995]<-xperm
x[,40000] = sort(x[,40000])
sampind<-sample(indind,250)
xsel<-x[sampind,40000]
xperm<-sample(xsel)
x[sampind,40000]<-xperm
x[,40005] = sort(x[,40005])
sampind<-sample(indind,250)
xsel<-x[sampind,40005]
xperm<-sample(xsel)
x[sampind,40005]<-xperm
x[,40010] = sort(x[,40010])
sampind<-sample(indind,250)
xsel<-x[sampind,40010]
xperm<-sample(xsel)
x[sampind,40010]<-xperm

true<-c(990,995,1000,1005,1010,
9990,9995,10000,10005,10010,
19990,19995,20000,20005,20010,
29990,29995,30000,30005,30010,
39990,39995,40000,40005,40010)

##########
# Lasso and elastic net analyses with cross validation
##########

#Lasso
cvmseLasso=cv.glmnet(x,ytot)
resmat[i,1]<-min(cvmseLasso$cvm)
resmat[i,2]<-cvmseLasso$cvm[which(cvmseLasso$lambda==cvmseLasso$lambda.1se)]

Lassopick<-which(coef(cvmseLasso,s="lambda.1se")>0)-1
Lassopick<-Lassopick[which(Lassopick>0)]
Lassotrue<-intersect(Lassopick,true)
nLassotrue<-length(Lassotrue)
nLassofalse<-length(Lassopick)-length(Lassotrue)


Lassopickmin<-which(coef(cvmseLasso,s="lambda.min")>0)-1
Lassopickmin<-Lassopickmin[which(Lassopickmin>0)]
Lassotruemin<-intersect(Lassopickmin,true)
nLassotruemin<-length(Lassotruemin)
```

```
nLassofalsemin<-length(Lassopickmin)-length(Lassotruemin)

# Elastic net – alpha 0.75
cvmseEN075=cv.glmnet(x,ytot,alpha=0.75)
resmat[i,3]<-min(cvmseEN075$cvm)
resmat[i,4]<-cvmseEN075$cvm[which(cvmseEN075$lambda==cvmseEN075$lambda.1se)]

EN075pick<-which(coef(cvmseEN075,s="lambda.1se")>0)-1
EN075pick<-EN075pick[which(EN075pick>0)]
EN075true<-intersect(EN075pick,true)
nEN075true<-length(EN075true)
nEN075false<-length(EN075pick)-length(EN075true)

EN075pickmin<-which(coef(cvmseEN075,s="lambda.min")>0)-1
EN075pickmin<-EN075pickmin[which(EN075pickmin>0)]
EN075truemin<-intersect(EN075pickmin,true)
nEN075truemin<-length(EN075truemin)
nEN075falsemin<-length(EN075pickmin)-length(EN075truemin)

# Elastic net – alpha 0.5
cvmseEN05=cv.glmnet(x,ytot,alpha=0.5)
resmat[i,5]<-min(cvmseEN05$cvm)
resmat[i,6]<-cvmseEN05$cvm[which(cvmseEN05$lambda==cvmseEN05$lambda.1se)]

EN05pick<-which(coef(cvmseEN05,s="lambda.1se")>0)-1
EN05pick<-EN05pick[which(EN05pick>0)]
EN05true<-intersect(EN05pick,true)
nEN05true<-length(EN05true)
nEN05false<-length(EN05pick)-length(EN05true)

EN05pickmin<-which(coef(cvmseEN05,s="lambda.min")>0)-1
EN05pickmin<-EN05pickmin[which(EN05pickmin>0)]
EN05truemin<-intersect(EN05pickmin,true)
nEN05truemin<-length(EN05truemin)
nEN05falsemin<-length(EN05pickmin)-length(EN05truemin)

# Elastic net – alpha 0.4
cvmseEN04<-cv.glmnet(x,ytot,alpha=0.4)
resmat[i,7]<-min(cvmseEN04$cvm)
resmat[i,8]<-cvmseEN04$nzero[which(cvmseEN04$lambda==cvmseEN04$lambda.1se)]

EN04pick<-which(coef(cvmseEN04,s="lambda.1se")>0)-1
EN04pick<-EN04pick[which(EN04pick>0)]
EN04true<-intersect(EN04pick,true)
nEN04true<-length(EN04true)
nEN04false<-length(EN04pick)-length(EN04true)

EN04pickmin<-which(coef(cvmseEN04,s="lambda.min")>0)-1
EN04pickmin<-EN04pickmin[which(EN04pickmin>0)]
EN04truemin<-intersect(EN04pickmin,true)
nEN04truemin<-length(EN04truemin)
nEN04falsemin<-length(EN04pickmin)-length(EN04truemin)
```

```
# Elastic net – alpha 0.3
cvmseEN03=cv.glmnet(x,ytot,alpha=0.3)
resmat[i,9]<-min(cvmseEN03$cvm)
resmat[i,10]<-cvmseEN03$cvm[which(cvmseEN03$lambda==cvmseEN03$lambda.1se)]

EN03pick<-which(coef(cvmseEN03,s="lambda.1se")>0)-1
EN03pick<-EN03pick[which(EN03pick>0)]
EN03true<-intersect(EN03pick,true)
nEN03true<-length(EN03true)
nEN03false<-length(EN03pick)-length(EN03true)

EN03pickmin<-which(coef(cvmseEN03,s="lambda.min")>0)-1
EN03pickmin<-EN03pickmin[which(EN03pickmin>0)]
EN03truemin<-intersect(EN03pickmin,true)
nEN03truemin<-length(EN03truemin)
nEN03falsemin<-length(EN03pickmin)-length(EN03truemin)

# Elastic net – alpha 0.2
cvmseEN02=cv.glmnet(x,ytot,alpha=0.2)
resmat[i,11]<-min(cvmseEN02$cvm)
resmat[i,12]<-cvmseEN02$nzero[which(cvmseEN02$lambda==cvmseEN02$lambda.1se)]

EN02pick<-which(coef(cvmseEN02,s="lambda.1se")>0)-1
EN02pick<-EN02pick[which(EN02pick>0)]
EN02true<-intersect(EN02pick,true)
nEN02true<-length(EN02true)
nEN02false<-length(EN02pick)-length(EN02true)

EN02pickmin<-which(coef(cvmseEN02,s="lambda.min")>0)-1
EN02pickmin<-EN02pickmin[which(EN02pickmin>0)]
EN02truemin<-intersect(EN02pickmin,true)
nEN02truemin<-length(EN02truemin)
nEN02falsemin<-length(EN02pickmin)-length(EN02truemin)

# Elastic net – alpha 0.15
cvmseEN015=cv.glmnet(x,ytot,alpha=0.15)
resmat[i,13]<-min(cvmseEN015$cvm)
resmat[i,14]<-cvmseEN015$nzero[which(cvmseEN015$lambda==cvmseEN015$lambda.1se)]

EN015pick<-which(coef(cvmseEN015,s="lambda.1se")>0)-1
EN015pick<-EN015pick[which(EN015pick>0)]
EN015true<-intersect(EN015pick,true)
nEN015true<-length(EN015true)
nEN015false<-length(EN015pick)-length(EN015true)

EN015pickmin<-which(coef(cvmseEN015,s="lambda.min")>0)-1
EN015pickmin<-EN015pickmin[which(EN015pickmin>0)]
EN015truemin<-intersect(EN015pickmin,true)
nEN015truemin<-length(EN015truemin)
nEN015falsemin<-length(EN015pickmin)-length(EN015truemin)

# Elastic net – alpha 0.1
cvmseEN01=cv.glmnet(x,ytot,alpha=0.1)
```

```
resmat[i,15]<-min(cvmseEN01$cvm)
resmat[i,16]<-cvmseEN01$cvm[which(cvmseEN01$lambda==cvmseEN01$lambda.1se)]

EN01pick<-which(coef(cvmseEN01,s="lambda.1se")>0)-1
EN01pick<-EN01pick[which(EN01pick>0)]
EN01true<-intersect(EN01pick,true)
nEN01true<-length(EN01true)
nEN01false<-length(EN01pick)-length(EN01true)

EN01pickmin<-which(coef(cvmseEN01,s="lambda.min")>0)-1
EN01pickmin<-EN01pickmin[which(EN01pickmin>0)]
EN01truemin<-intersect(EN01pickmin,true)
nEN01truemin<-length(EN01truemin)
nEN01falsemin<-length(EN01pickmin)-length(EN01truemin)

# Elastic net – alpha 0.05
cvmseEN005=cv.glmnet(x,ytot,alpha=0.05)
resmat[i,17]<-min(cvmseEN005$cvm)
resmat[i,18]<-cvmseEN005$cvm[which(cvmseEN005$lambda==cvmseEN005$lambda.1se)]

EN005pick<-which(coef(cvmseEN005,s="lambda.1se")>0)-1
EN005pick<-EN005pick[which(EN005pick>0)]
EN005true<-intersect(EN005pick,true)
nEN005true<-length(EN005true)
nEN005false<-length(EN005pick)-length(EN005true)

EN005pickmin<-which(coef(cvmseEN005,s="lambda.min")>0)-1
EN005pickmin<-EN005pickmin[which(EN005pickmin>0)]
EN005truemin<-intersect(EN005pickmin,true)
nEN005truemin<-length(EN005truemin)
nEN005falsemin<-length(EN005pickmin)-length(EN005truemin)

# Elastic net – alpha 0.01
cvmseEN001=cv.glmnet(x,ytot,alpha=0.01)
resmat[i,19]<-min(cvmseEN001$cvm)
resmat[i,20]<-cvmseEN001$cvm[which(cvmseEN001$lambda==cvmseEN001$lambda.1se)]

EN001pick<-which(coef(cvmseEN001,s="lambda.1se")>0)-1
EN001pick<-EN001pick[which(EN001pick>0)]
EN001true<-intersect(EN001pick,true)
nEN001true<-length(EN001true)
nEN001false<-length(EN001pick)-length(EN001true)

EN001pickmin<-which(coef(cvmseEN001,s="lambda.min")>0)-1
EN001pickmin<-EN001pickmin[which(EN001pickmin>0)]
EN001truemin<-intersect(EN001pickmin,true)
nEN001truemin<-length(EN001truemin)
nEN001falsemin<-length(EN001pickmin)-length(EN001truemin)

resmat1se[i,]<-c(nLassotrue,nLassofalse,nEN075true,nEN075false,nEN05true,nEN05false,
                 nEN04true,nEN04false,nEN03true,nEN03false,nEN02true,nEN02false,
                 nEN015true,nEN015false,nEN01true,nEN01false,nEN005true,nEN005false,
                 nEN001true,nEN001false)
```

```
resmatmin[i,]<-c(nLassotruemin,nLassofalsemin,nEN075truemin,nEN075falsemin,
                 nEN05truemin,nEN05falsemin,nEN04truemin,nEN04falsemin,
                 nEN03truemin,nEN03falsemin,nEN02truemin,nEN02falsemin,
                 nEN015truemin,nEN015falsemin,nEN01truemin,nEN01falsemin,
                 nEN005truemin,nEN005falsemin,nEN001truemin,nEN001falsemin)
}

dput(resmat,"SimResMixedLDMSE.txt")
dput(resmat1se," SimResMixedLD1se.txt")
dput(resmatmin," SimResMixedLDmin.txt")

######################################################
# High LD setting
######################################################
rm(list=ls(all=TRUE))

library(Matrix)
library(glmnet)

resmat1se<-matrix(NA,nrow=100,ncol=20)
resmatmin<-matrix(NA,nrow=100,ncol=20)
resmat<-matrix(NA,nrow=100,ncol=20)

# 100 replicates of the HighLD setting
for(i in 1:100){

# generation of simulated data
ylow=rnorm(500,-5,1)
yhigh=rnorm(500,5,1)
ytot=c(ylow,yhigh) # phenotype

x1=Matrix(rbinom(5e+07,1,0.5),nrow=1000,ncol=50000)
x2=Matrix(rbinom(5e+07,1,0.5),nrow=1000,ncol=50000)

x<- x1+x2 # genotypes with 0-1-2 coding


x[,990] = sort(x[,990])
x[,995] = sort(x[,995])
x[,1000] = sort(x[,1000])
x[,1005] = sort(x[,1005])
x[,1010] = sort(x[,1010])

x[,9990] = sort(x[,9990])
x[,9995] = sort(x[,9995])
x[,10000] = sort(x[,10000])
x[,10005] = sort(x[,10005])
x[,10010] = sort(x[,10010])

x[,19990] = sort(x[,19990])
x[,19995] = sort(x[,19995])
x[,20000] = sort(x[,20000])
x[,20005] = sort(x[,20005])
```

```
x[,20010] = sort(x[,20010])

x[,29990] = sort(x[,29990])
x[,29995] = sort(x[,29995])
x[,30000] = sort(x[,30000])
x[,30005] = sort(x[,30005])
x[,30010] = sort(x[,30010])

x[,39990] = sort(x[,39990])
x[,39995] = sort(x[,39995])
x[,40000] = sort(x[,40000])
x[,40005] = sort(x[,40005])
x[,40010] = sort(x[,40010])

true<-c(990,995,1000,1005,1010,
9990,9995,10000,10005,10010,
19990,19995,20000,20005,20010,
29990,29995,30000,30005,30010,
39990,39995,40000,40005,40010)

##########
# Lasso and elastic net analyses with cross validation
##########

#Lasso
cvmseLasso=cv.glmnet(x,ytot)
resmat[i,1]<-min(cvmseLasso$cvm)
resmat[i,2]<-cvmseLasso$cvm[which(cvmseLasso$lambda==cvmseLasso$lambda.1se)]

Lassopick<-which(coef(cvmseLasso,s="lambda.1se")>0)-1
Lassopick<-Lassopick[which(Lassopick>0)]
Lassotrue<-intersect(Lassopick,true)
nLassotrue<-length(Lassotrue)
nLassofalse<-length(Lassopick)-length(Lassotrue)


Lassopickmin<-which(coef(cvmseLasso,s="lambda.min")>0)-1
Lassopickmin<-Lassopickmin[which(Lassopickmin>0)]
Lassotruemin<-intersect(Lassopickmin,true)
nLassotruemin<-length(Lassotruemin)
nLassofalsemin<-length(Lassopickmin)-length(Lassotruemin)

# Elastic net – alpha 0.75
cvmseEN075=cv.glmnet(x,ytot,alpha=0.75)
resmat[i,3]<-min(cvmseEN075$cvm)
resmat[i,4]<-cvmseEN075$cvm[which(cvmseEN075$lambda==cvmseEN075$lambda.1se)]

EN075pick<-which(coef(cvmseEN075,s="lambda.1se")>0)-1
EN075pick<-EN075pick[which(EN075pick>0)]
EN075true<-intersect(EN075pick,true)
nEN075true<-length(EN075true)
nEN075false<-length(EN075pick)-length(EN075true)
```

```
EN075pickmin<-which(coef(cvmseEN075,s="lambda.min")>0)-1
EN075pickmin<-EN075pickmin[which(EN075pickmin>0)]
EN075truemin<-intersect(EN075pickmin,true)
nEN075truemin<-length(EN075truemin)
nEN075falsemin<-length(EN075pickmin)-length(EN075truemin)

# Elastic net – alpha 0.5
cvmseEN05=cv.glmnet(x,ytot,alpha=0.5)
resmat[i,5]<-min(cvmseEN05$cvm)
resmat[i,6]<-cvmseEN05$cvm[which(cvmseEN05$lambda==cvmseEN05$lambda.1se)]

EN05pick<-which(coef(cvmseEN05,s="lambda.1se")>0)-1
EN05pick<-EN05pick[which(EN05pick>0)]
EN05true<-intersect(EN05pick,true)
nEN05true<-length(EN05true)
nEN05false<-length(EN05pick)-length(EN05true)

EN05pickmin<-which(coef(cvmseEN05,s="lambda.min")>0)-1
EN05pickmin<-EN05pickmin[which(EN05pickmin>0)]
EN05truemin<-intersect(EN05pickmin,true)
nEN05truemin<-length(EN05truemin)
nEN05falsemin<-length(EN05pickmin)-length(EN05truemin)

# Elastic net – alpha 0.4
cvmseEN04<-cv.glmnet(x,ytot,alpha=0.4)
resmat[i,7]<-min(cvmseEN04$cvm)
resmat[i,8]<-cvmseEN04$nzero[which(cvmseEN04$lambda==cvmseEN04$lambda.1se)]

EN04pick<-which(coef(cvmseEN04,s="lambda.1se")>0)-1
EN04pick<-EN04pick[which(EN04pick>0)]
EN04true<-intersect(EN04pick,true)
nEN04true<-length(EN04true)
nEN04false<-length(EN04pick)-length(EN04true)

EN04pickmin<-which(coef(cvmseEN04,s="lambda.min")>0)-1
EN04pickmin<-EN04pickmin[which(EN04pickmin>0)]
EN04truemin<-intersect(EN04pickmin,true)
nEN04truemin<-length(EN04truemin)
nEN04falsemin<-length(EN04pickmin)-length(EN04truemin)

# Elastic net – alpha 0.3
cvmseEN03=cv.glmnet(x,ytot,alpha=0.3)
resmat[i,9]<-min(cvmseEN03$cvm)
resmat[i,10]<-cvmseEN03$cvm[which(cvmseEN03$lambda==cvmseEN03$lambda.1se)]

EN03pick<-which(coef(cvmseEN03,s="lambda.1se")>0)-1
EN03pick<-EN03pick[which(EN03pick>0)]
EN03true<-intersect(EN03pick,true)
nEN03true<-length(EN03true)
nEN03false<-length(EN03pick)-length(EN03true)

EN03pickmin<-which(coef(cvmseEN03,s="lambda.min")>0)-1
EN03pickmin<-EN03pickmin[which(EN03pickmin>0)]
```

```
EN03truemin<-intersect(EN03pickmin,true)
nEN03truemin<-length(EN03truemin)
nEN03falsemin<-length(EN03pickmin)-length(EN03truemin)

# Elastic net – alpha 0.2
cvmseEN02=cv.glmnet(x,ytot,alpha=0.2)
resmat[i,11]<-min(cvmseEN02$cvm)
resmat[i,12]<-cvmseEN02$nzero[which(cvmseEN02$lambda==cvmseEN02$lambda.1se)]

EN02pick<-which(coef(cvmseEN02,s="lambda.1se")>0)-1
EN02pick<-EN02pick[which(EN02pick>0)]
EN02true<-intersect(EN02pick,true)
nEN02true<-length(EN02true)
nEN02false<-length(EN02pick)-length(EN02true)

EN02pickmin<-which(coef(cvmseEN02,s="lambda.min")>0)-1
EN02pickmin<-EN02pickmin[which(EN02pickmin>0)]
EN02truemin<-intersect(EN02pickmin,true)
nEN02truemin<-length(EN02truemin)
nEN02falsemin<-length(EN02pickmin)-length(EN02truemin)

# Elastic net – alpha 0.15
cvmseEN015=cv.glmnet(x,ytot,alpha=0.15)
resmat[i,13]<-min(cvmseEN015$cvm)
resmat[i,14]<-cvmseEN015$nzero[which(cvmseEN015$lambda==cvmseEN015$lambda.1se)]

EN015pick<-which(coef(cvmseEN015,s="lambda.1se")>0)-1
EN015pick<-EN015pick[which(EN015pick>0)]
EN015true<-intersect(EN015pick,true)
nEN015true<-length(EN015true)
nEN015false<-length(EN015pick)-length(EN015true)

EN015pickmin<-which(coef(cvmseEN015,s="lambda.min")>0)-1
EN015pickmin<-EN015pickmin[which(EN015pickmin>0)]
EN015truemin<-intersect(EN015pickmin,true)
nEN015truemin<-length(EN015truemin)
nEN015falsemin<-length(EN015pickmin)-length(EN015truemin)

# Elastic net – alpha 0.1
cvmseEN01=cv.glmnet(x,ytot,alpha=0.1)
resmat[i,15]<-min(cvmseEN01$cvm)
resmat[i,16]<-cvmseEN01$cvm[which(cvmseEN01$lambda==cvmseEN01$lambda.1se)]

EN01pick<-which(coef(cvmseEN01,s="lambda.1se")>0)-1
EN01pick<-EN01pick[which(EN01pick>0)]
EN01true<-intersect(EN01pick,true)
nEN01true<-length(EN01true)
nEN01false<-length(EN01pick)-length(EN01true)

EN01pickmin<-which(coef(cvmseEN01,s="lambda.min")>0)-1
EN01pickmin<-EN01pickmin[which(EN01pickmin>0)]
EN01truemin<-intersect(EN01pickmin,true)
nEN01truemin<-length(EN01truemin)
```

```
nEN01falsemin<-length(EN01pickmin)-length(EN01truemin)

# Elastic net – alpha 0.05
cvmseEN005=cv.glmnet(x,ytot,alpha=0.05)
resmat[i,17]<-min(cvmseEN005$cvm)
resmat[i,18]<-cvmseEN005$cvm[which(cvmseEN005$lambda==cvmseEN005$lambda.1se)]

EN005pick<-which(coef(cvmseEN005,s="lambda.1se")>0)-1
EN005pick<-EN005pick[which(EN005pick>0)]
EN005true<-intersect(EN005pick,true)
nEN005true<-length(EN005true)
nEN005false<-length(EN005pick)-length(EN005true)

EN005pickmin<-which(coef(cvmseEN005,s="lambda.min")>0)-1
EN005pickmin<-EN005pickmin[which(EN005pickmin>0)]
EN005truemin<-intersect(EN005pickmin,true)
nEN005truemin<-length(EN005truemin)
nEN005falsemin<-length(EN005pickmin)-length(EN005truemin)

# Elastic net – alpha 0.01
cvmseEN001=cv.glmnet(x,ytot,alpha=0.01)
resmat[i,19]<-min(cvmseEN001$cvm)
resmat[i,20]<-cvmseEN001$cvm[which(cvmseEN001$lambda==cvmseEN001$lambda.1se)]

EN001pick<-which(coef(cvmseEN001,s="lambda.1se")>0)-1
EN001pick<-EN001pick[which(EN001pick>0)]
EN001true<-intersect(EN001pick,true)
nEN001true<-length(EN001true)
nEN001false<-length(EN001pick)-length(EN001true)

EN001pickmin<-which(coef(cvmseEN001,s="lambda.min")>0)-1
EN001pickmin<-EN001pickmin[which(EN001pickmin>0)]
EN001truemin<-intersect(EN001pickmin,true)
nEN001truemin<-length(EN001truemin)
nEN001falsemin<-length(EN001pickmin)-length(EN001truemin)

resmat1se[i,]<-c(nLassotrue,nLassofalse,nEN075true,nEN075false,nEN05true,nEN05false,
                 nEN04true,nEN04false,nEN03true,nEN03false,nEN02true,nEN02false,
                 nEN015true,nEN015false,nEN01true,nEN01false,nEN005true,nEN005false,
                 nEN001true,nEN001false)
resmatmin[i,]<-c(nLassotruemin,nLassofalsemin,nEN075truemin,nEN075falsemin,
                 nEN05truemin,nEN05falsemin,nEN04truemin,nEN04falsemin,
                 nEN03truemin,nEN03falsemin,nEN02truemin,nEN02falsemin,
                 nEN015truemin,nEN015falsemin,nEN01truemin,nEN01falsemin,
                 nEN005truemin,nEN005falsemin,nEN001truemin,nEN001falsemin)
}

dput(resmat,"SimResHighLDMSE.txt")
dput(resmat1se," SimResHighLD1se.txt")
dput(resmatmin," SimResHighLDmin.txt")
```
